# *TPX2* overexpression promotes sensitivity to dasatinib in breast cancer by activating YAP transcriptional signaling

**DOI:** 10.1002/1878-0261.13602

**Published:** 2024-02-15

**Authors:** Carlos Marugán, Natalia Sanz‐Gómez, Beatriz Ortigosa, Ana Monfort‐Vengut, Cristina Bertinetti, Ana Teijo, Marta González, Alicia Alonso de la Vega, María José Lallena, Gema Moreno‐Bueno, Guillermo de Cárcer

**Affiliations:** ^1^ Cell Cycle & Cancer Biomarkers Laboratory, Cancer Department Instituto de Investigaciones Biomédicas Sols‐Morreale (IIBM) CSIC‐UAM Madrid Spain; ^2^ Discovery Chemistry Research and Technology Eli Lilly and Company Madrid Spain; ^3^ Translational Cancer Research Laboratory, Cancer Department Instituto de Investigaciones Biomédicas Alberto Sols‐Morreale (IIBM) CSIC‐UAM Madrid Spain; ^4^ Pathology Department MD Anderson Cancer Center Madrid Spain; ^5^ MD Anderson International Foundation Madrid Spain; ^6^ Biomedical Cancer Research Network (CIBERONC) Madrid Spain; ^7^ CSIC Conexión‐Cáncer Hub (https://conexion‐cancer.csic.es)

**Keywords:** chromosomal instability, dasatinib, Hippo‐YAP/TAZ, mitosis, SFK kinases, *TPX2*

## Abstract

Chromosomal instability (CIN) is a hallmark of cancer aggressiveness, providing genetic plasticity and tumor heterogeneity that allows the tumor to evolve and adapt to stress conditions. CIN is considered a cancer therapeutic biomarker because healthy cells do not exhibit CIN. Despite recent efforts to identify therapeutic strategies related to CIN, the results obtained have been very limited. CIN is characterized by a genetic signature where a collection of genes, mostly mitotic regulators, are overexpressed in CIN‐positive tumors, providing aggressiveness and poor prognosis. We attempted to identify new therapeutic strategies related to CIN genes by performing a drug screen, using cells that individually express CIN‐associated genes in an inducible manner. We find that the overexpression of targeting protein for Xklp2 (*TPX2*) enhances sensitivity to the proto‐oncogene c‐Src (SRC) inhibitor dasatinib due to activation of the Yes‐associated protein 1 (YAP) pathway. Furthermore, using breast cancer data from The Cancer Genome Atlas (TCGA) and a cohort of cancer‐derived patient samples, we find that both *TPX2* overexpression and YAP activation are present in a significant percentage of cancer tumor samples and are associated with poor prognosis; therefore, they are putative biomarkers for selection for dasatinib therapy.

## Graphical abstract

Previous efforts trying to find a therapeutic strategy using chromosomal instability (CIN) as a biomarker did not yield promising results. We screened a drug library using breast cancer cells overexpressing individual genes, closely associated with CIN, in an inducible manner. We found that overexpression of the CIN‐associated gene *TPX2* provides sensitivity to the SRC inhibitor dasatinib, due to an increase in YAP/TAZ transcriptional signaling.

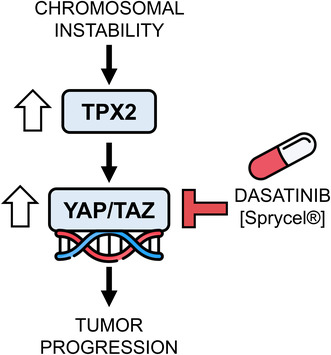

**Abbreviations**
ANOVA: analysis of variance
AUC: area under the curve
CIN: chromosomal instability
CNA: copy number alteration
CRC: concentration‐response curves
CTRP: cancer therapeutics response portal
DAPI: 4′,6‐diamidino‐2‐phenylindole
DMSO: dimethyl sulfoxide
DOX: doxycycline
DPBQ: 8‐azaguanine and 2,3‐diphenylbenzo[g]quinoxaline‐5,10‐dione
EDTA: ethylenediaminetetraacetic acid
ER: estrogen receptor
FDR: false discovery rate
GSEA: gene set enrichment analysis
HER2: human epidermal growth factor receptor 2
HR: homologous recombination
IHC: immunohistochemistry
METABRIC: Molecular Taxonomy of Breast Cancer International Consortium
NCCN: National Comprehensive Cancer Network
NES: normalized enrichment score
NHEJ: non‐homologous end joining
NIH: National Institutes of Health
PBS: phosphate‐buffered saline buffer
qRT‐PCR: real‐time quantitative reverse transcription PCR
RF: relapse free
RIPA: radioimmunoprecipitation assay buffer
rtTA: reverse transcriptional activator
SDS: sodium dodecyl sulfate
SFK: SRC family kinases
TCGA: The Cancer Genome Atlas
TPM: transcripts per million

## 1 Introduction

Chromosomal instability (CIN) is defined by a high rate of chromosome mis‐segregation leading to chromosomal copy number alterations (CNAs) affecting either whole chromosomes or chromosome arm fragments [1, 2]. These CNAs ultimately result in an uneven distribution of DNA after cell division leading to aneuploidy [3]. In the context of cancer, CIN is a source of phenotypic variation that generates heterogeneity at the chromosome copy number and gene dosage level, leading to tumor progression, aggressiveness, metastasis, recurrence, and drug resistance [3, 4, 5]. CIN is a common feature of aggressiveness in breast cancer, being present in an 89% of invasive breast cancer cases, suggesting that it has potential application in breast cancer diagnosis and treatment [6].

CIN is rare in normal tissues but common in cancer, with 60–80% of human tumors exhibiting CIN [5, 7]. This suggests a therapeutic opportunity considering CIN as a biomarker for tumor cells, since any therapy targeting CIN would not affect healthy cells [8], and several studies evaluated the possibility of linking drug sensitivity and CIN. By screening aneuploid cells against a drug library, AICAR (AMPK activator) and 17‐AAG (Hsp90 inhibitor) were identified as compounds able to specifically inhibit the proliferation of positive CIN cells [9]. Similarly, by an *in silico* data correlation analysis, comparing drug sensitivity for around 45 000 chemicals and ploidy data from the NCI‐60 database, 8‐azaguanine and 2,3‐diphenylbenzo[g]quinoxaline‐5,10‐dione (DPBQ) were identified as aneuploid‐selective killing compounds [10]. Despite these initial attempts, no more advances are significant in this regard; consequently, new strategies for targeting CIN cancer cells are needed.

CIN is defined by a particular genetic signature obtained by correlating the expression of more than 10 000 genes versus a score of functional aneuploidy, as a surrogate value of CIN [6]. The top‐listed genes with the highest CIN score constitute the CIN70 expression signature. Overexpression of the CIN70 signature is associated with poor clinical outcomes in several cancers. In addition, other studies identified similar CIN signatures for a variety of cancers, correlating with increased tumoral aggressiveness [11, 12, 13, 14, 15] and metastasis capacity [16]. The CIN signatures are particularly enriched in mitotic genes intimately linked to ensure proper chromosome segregation in each cell cycle [17, 18]. The most representative CIN70 signature gene is *TPX2*, an essential mitotic gene participating in the spindle assembly by activating the Aurora‐A kinase [19, 20, 21, 22, 23]. *TPX2* is overexpressed in a wide variety of tumors, and this is a hallmark of poor prognosis [17, 24, 25]. In breast cancer, it is common to find genetic amplification of the *TPX2* locus (20q11.21), that might modulate the proliferation of high CIN tumors [26]. Genetic depletion of *TPX2* leads to severe mitotic aberrations indicating its essentiality in cell proliferation [27]. *TPX2* overexpression also leads to mitotic changes that can cause CIN and compromise cell viability [28].

The Hippo‐YAP/TAZ signaling axis is an essential pathway regulating cell proliferation, cell plasticity, and organ growth during animal development. It is modulated by several inputs such as cell polarity signaling, cell–cell adhesion, cell contact inhibition, mechanotransduction, etc [29, 30, 31]. In the context of cancer, the Hippo‐YAP/TAZ pathway is often deregulated, promoting adaptation and proliferation capacity to cancer cells [32, 33], and is a major sensor for adapting to elevated CIN levels [34, 35]. Indeed, elevated *YAP* activity correlates with CIN levels in cancer samples [36, 37, 38]. The Hippo‐YAP/TAZ pathway is considered a bona fide target for cancer therapy, especially by inhibition of the YAP/TAZ transcription activity [32, 33]. In this regard, verteporfin was identified as an efficient inhibitor of the YAP/TAZ binding to the TEAD transcription factors [39], leading to an efficient downregulation of YAP/TAZ target genes. Other attempts to find YAP/TAZ inhibitors were done by screening drugs that inhibit the YAP/TAZ nuclear translocation, finding dasatinib, statins, and pazopanib as positive hits in breast cancer cell lines [40, 41].

The specific sensitivity of YAP/TAZ signaling to dasatinib is based on the close interaction between YAP and the SRC‐family kinases (SFKs) signaling. The SFK family is comprised of 11 kinases (BLK, BRK, FGR, FYN, FRK, HCK, LCK, LYN, SRC, SRM, and YES) [42]. YAP gets its name from “YES‐Associated Protein 1” because it binds to the SH3 domain of YES kinase [43]. SFKs activate YAP/TAZ either by direct YAP phosphorylation or by repressing the Hippo pathway by LATS1/2 kinases phosphorylation [44]. The interaction with SFKs is essential for YAP signaling activation in a wide variety of tumoral cells [44, 45, 46, 47, 48, 49].

In this work, we screened MDA‐MB‐453 breast cancer cells that individually overexpress a collection of CIN‐related genes (*BIRC5*, *CCNB1*, *CCNB2*, *ECT2*, *HEC1*, *MAD2*, *PRC1*, *PTTG1*, and *TPX2*) in an inducible manner by the TET‐ON system, against a panel of 60 drugs already being used in the clinic or in advanced‐stage clinical trials, which inhibit representative signaling pathways in cancer (Table S1). We found that *TPX2* overexpression provides an enhanced sensitivity towards the SRC inhibitor dasatinib, sustained on an increased YAP/TAZ signaling dependency. Moreover, after exploring the Molecular Taxonomy of Breast Cancer International Consortium (METABRIC) project, and a cohort of breast cancer‐derived samples, we show that *TPX2* overexpression and YAP activation are coincident biomarkers in a significant proportion of aggressive breast cancer samples, suggesting dasatinib as an alternative therapeutic avenue.

## 2 Materials and methods

### 2.1 Cell line culture

MDA‐MB‐453 (RRID:CVCL_0418), ZR‐75‐1 (RRID:CVCL_0588), MDA‐MB‐361 (RRID:CVCL_0620), HCC‐1937 (RRID:CVCL_0290), and HEK293 (RRID:CVCL_0063) were obtained from the ATCC repository and cultured in DMEM 10% FBS (MDA‐MB‐453, HCC‐1937 and HEK293), DMEM 20% FBS (MDA‐MB‐361) and RPMI‐1640 10% FBS (ZR‐75‐1), in a humid incubator with 5% CO_2_ atmosphere and 37 °C. Cultures were periodically tested for Mycoplasma contamination, and authenticated by the GenePrint® 10 System (Promega, Madison, WI, USA), and data were analyzed using genemapper® id‐x v1.2 software (Applied Biosystems, Waltham, MA, USA).

### 2.2 CIN‐related cDNAs cloning

The *BIRC5*, *CCNB1*, *CCNB2*, *ECT2*, *HEC1*, *MAD2*, *PRC1*, *PTTG1*, and *TPX2* cDNAs were obtained from the Mammalian Gene Collection repository, amplified by the Expand High Fidelity PCR system (Roche, Basel, Switzerland) (primers depicted in Table S2), and cloned into pENTR/D‐TOPO plasmid. The final inducible expression plasmids were generated in the pLenti‐CMVtight‐Hygro‐DEST by the Gateway LR‐Clonase II Enzyme Mix system (Invitrogen, Waltham, MA, USA). All cDNA amplification and cloning steps were validated by DNA sequencing.

### 2.3 Lentiviral particle production and generation of the TET‐ON inducible expression system cell line

HEK293 cells were transfected with a mixture of the 3rd generation lentiviral packaging plasmids containing: 2.5 μg of Rev (pRSV‐Rev, Addgene #12253; Addgene, Watertown, MA, USA), 6.5 μg of Gag and Pol (pMDLg/pRRE, Addgene #12251), and 3.5 μg of VSV‐G envelope expressing plasmid (pMD2.G, Addgene #12259), and either 10 μg of the Tet‐On‐3G transactivator (pLVX Tet3G, Clontech, Mountain View, CA, USA) or 10 μg of the pLenti‐CMVtight‐Hygro‐DEST plasmid with each cDNA of interest, and 50 μL Lipofectamine 2000 (Invitrogen) in Opti‐MEM (Gibco). Viral supernatants were retrieved after 24, 48, and 72 h, cleared through a 0.45 μm pore‐size filter and stored at −80 °C.

MDA‐MB‐453 cells were firstly transduced with the pLVX Tet3G viral particle for the rtTA transactivator expression, and selected in 400 μg·mL^−1^ of geneticin (Gibco). rtTA‐expressing cells were then infected with the pLenti‐CMVtight DEST plasmids expressing each cDNA of interest, and selected in 50 μg·mL^−1^ of Hygromycin B. Cell stocks were amplified and stored in liquid nitrogen. MDA‐MB‐453 Tet‐ON cells were grown in the presence of 0.01, 0.1, or 1.0 μg·mL^−1^ of doxycycline (DOX) to test each cDNA expression.

### 2.4 Standard of care drug library screening

A total of 60 kinase inhibitors, including staurosporine as a positive control, were used for the drug screen experiments. These 60 inhibitors represent a collection of standards of care for many different signaling pathways and cellular processes (Table S1). The MDA‐MB‐453 Tet‐ON cell lines were dispensed in two 384‐well Poly‐d‐lysine Biocoat plates, per cDNA of interest. For cDNA induction, DOX was added to one replicate plate at a final concentration of 0.1 μg·mL^−1^, and plain growth media to the other replicate plate. After overnight incubation, 10 serial dilutions of the compound library (from 20 to 0.001 μm) were added to each plate, and cells were then allowed to grow for two population‐doubling times. DOX and drug treatment was renewed every 48 h.

After gene induction and drug incubation, cells were fixed in 70% cold ethanol and stained with 0.4 μg·mL^−1^ of DAPI. The number of cells in each well was counted with Acumen eX3 (TTP LabTech, Cambridge, MA, USA), normalized versus the DMSO control wells, and then the ratio noDOX/+DOX was calculated to evaluate the effect of each drug. prism (GraphPad, La Jolla, CA, USA) was used to generate and analyze the scatter plots. IC50s were calculated using genedata screener software (Genedata Screener, Basel, Switzerland) using the normalized cell number in each well.

### 2.5 Cell colony formation and drug treatments

MDA‐MB‐453, ZR‐75‐1, MDA‐MB‐361, and HCC‐1937 TET‐ON/*TPX2* cells were seeded in triplicates in 12 well plates. *TPX2* overexpression was induced by adding 0.01, 0.1, or 1.0 μg·mL^−1^ of DOX. The next day plates were treated with DMSO or the mentioned inhibitor concentration. DOX and inhibitors treatment were renewed every 2 to 3 days. After 12 to 14 days, the plates were fixed in 4% formaldehyde in PBS, stained with Giemsa, and scanned in an Epson V800 scanner. The colony area was quantified using image j (NIH, Bethesda, MD, USA) and its ColonyArea plugin [50].

### 2.6 Protein extraction and immunoblotting assays

Cells were lysed in RIPA buffer (37 mm NaCl, 0.5% NP‐40, 0.1% SDS, 1% Triton X‐100, 20 mm Tris–HCl, pH 7.4, 2 mm EDTA, 10% glycerol, supplemented with protease and phosphatase inhibitory cocktails (SIGMA‐Aldrich, Burlington, MA, USA)) on ice during 20 min, and protein lysates clarified by 30‐min centrifugation at 17 000 ***g*** Protein concentration was quantified using Pierce™ BCA Protein Assay kit (Thermo Fisher Scientific, Waltham, MA, USA). Proteins were separated in Novex™ 4–20% tris‐glycine acrylamide gels (Invitrogen) and transferred to nitrocellulose membranes (BioRad, Hercules, CA, USA). Blotted proteins were blocked in 5% non‐fat milk in PBS‐T (PBS with 0.05% Tween‐20) and probed with the corresponding primary antibody (Table S3). Secondary antibodies coupled to fluorescent IRDye680 were incubated for 45 min and scanned with the Odyssey Infrared Imaging System (Li‐Cor Biotechnology, Lincoln, NE, USA).

### 2.7 Flow cytometry cell cycle analysis

Cells were trypsinized, fixed with cold 70% ethanol, and resuspended in PBS‐T (PBS + 0.03% TritonX‐100). DNA was stained with 1 μg·mL^−1^ DAPI, and DNA profile data were retrieved using a FACSCantoII device and analyzed using facsdiva software (Becton Dickinson, Franklin Lakes, NJ, USA).

### 2.8 Cell immunofluorescence and YAP signal quantification

Cells were fixed in 4% methanol‐free formaldehyde (PolySciences, Warrington, PA, USA) in PBS, permeabilized with cold methanol, blocked with 10% fetal bovine serum in PBS‐T (PBS + 0.03% TritonX‐100), and incubated with primary antibodies against phospho‐histone H3‐Ser10 (Cell Signaling Technology #3377s; Cell Signaling Technologies, Danvers, MA, USA), total YAP (Santa Cruz Biotechnology #sc‐271134; Santa Cruz Biotechnology, Dallas, TX, USA), or active‐YAP (Abcam #ab205270; Abcam, Cambridge, UK) diluted in PBS‐T. A secondary antibody coupled to the Alexa488 dye (Invitrogen – Molecular Probes) was used. DNA was counterstained with 0.1 μg·mL^−1^ DAPI, and cells were finally mounted in glass slides using ProLong Diamond antifade mounting media (Thermo Fischer). Pictures were obtained using a NIKON 90‐eclipse microscope. YAP nuclear/cytoplasm ratio was calculated using the imagej Intensity Ratio Nuclei Cytoplasm tool (RRID:SCR_018573).

### 2.9 Quantitative Real‐Time PCR

Single‐gene qRT‐PCR analysis was done using FAM‐MGB TaqMan probes (Invitrogen) specific for *Cyr61* (Hs00155479_m1), *CTGF* (Hs01026927_g1), and *TEAD4* (Hs01125032_m1) genes. RNA was extracted with Trizol and column‐purified with the Absolutely RNA miniprep kit (Stratagene, La Jolla, CA, USA). Both cDNA synthesis and PCR amplification were done with the SuperScript III one‐step RT‐PCR system (Invitrogen). TaqMan probes for the housekeeping genes *ACTB* (Hs01060665_g1) or *HPRT1* (Hs02800695_m1) were used as normalization controls.

### 2.10 METABRIC *in silico* data analysis

Clinical and expression data of a cohort of 1536 patients were downloaded from the Molecular Taxonomy of Breast Cancer International Consortium (METABRIC) breast cancer project [51]. Patients were classified according to *TPX2* expression with a membership probability estimated by bootstrap [52]. Gene Set Enrichment Analysis (GSEA) was performed by comparing high versus low *TPX2* expressing samples. The top 25 terms of C6: Oncogenic Signature were analyzed. Additionally, we performed a GSEA with the TAZ/YAP signature [53]. FDR *q* < 0.05 was considered significant. Samples classified according to *TPX2* expression were also classified with the YAP/TAZ‐signature expression as in [54]. Briefly, after quantile normalization, a z‐score was calculated for the expression data. Genes included in the YAP/TAZ signature were extracted, and each sample's combined score was calculated as the sum of the individual expression z‐scores. Samples were then classified as having a low TAZ/YAP signature if the combined score was less than the combined mean, and as having a high TAZ/YAP signature if the combined score was greater than the combined mean. Contingency tables were analyzed by a Fisher's Exact Test.

### 2.11 Human breast cancer tumoral samples IHC analysis

A total of 99 paraffin‐embedded grade 3 Invasive Ductal Breast Carcinoma (IDBC), molecularly classified according to the NCCN breast carcinoma guidelines, were subjected to immunohistochemistry (IHC) analysis. Study procedures were carried out in accordance with the Declaration of Helsinki, as revised in 2008, and good clinical practice guidelines. Samples were obtained from the MD Anderson Foundation Biobank (record number B.0000745, ISCIII National Biobank Record‐Madrid) between 2003 and 2014, under the Ethical protocol number (MDA‐LEP‐2020‐01) approved on 29/04/2021. Written informed consent was obtained from all patients before enrollment. This study followed all the standard ethical procedures of the Spanish regulation (Ley de Investigación Orgánica Biomédica, 14 July 2007) and was approved by the ethic committees of the MD Anderson Cancer Center Madrid, (Madrid – Spain). The mean age of the patients was 59.6 ± 9.1 years. Non‐tumoral breast tissue was analyzed as an internal control. The study was approved by the local ethical committee from each institution, and complete written informed consent was obtained from all patients. Written informed consent was obtained from all patients before enrollment. The collected tumor samples were fixed in 4% formaldehyde and embedded in paraffin. Immunohistochemistry was done using TPX2 antibody (Abcam ab32795), phosphor‐Ser127‐YAP (Abcam ab76252), and total YAP (Santa Cruz Biotechnology sc‐271134). Tumor sections were deparaffinized using the BOND Dewax Solution (Leica Biosystems, Nussloch, Germany) and rehydrated passing through decreasing ethanol solutions. Immunostaining was performed on a BOND RXm autostainer using the BOND Epitope Retrieval Solutions and Polymer Refine Detection kit (Leica Biosystems). The slides were then stained with hematoxylin, and examined with the aperio eslide manager software (Leica Biosystems). Quantification scoring of each marker was done by evaluating staining intensity and the percentage of positive tumor cells was.

### 2.12 Statistical analysis and DepMap *in silico* data browsing

Statistical analysis was performed using prism software (GraphPad). The statistical significance was evaluated using either one‐way ANOVA, two‐way ANOVA, or correlation and linear regression. Data were plotted as mean ± SD or mean ± SEM. Probabilities of less than 0.05 were considered statistically significant: *P* < 0.05 (*); *P* < 0.01 (**); *P* < 0.001 (***); *P* < 0.0001 (****).

For tumor samples, statistical analysis was performed using spss Statistics 28.0.1.1 (SPSS Inc., Chicago, IL, USA). The chi‐square or Fisher's exact tests were used to test associations between categorical variables. All tests were two‐tailed, and 95% confidence intervals (CIs) were used. Values of *P* < 0.05 were considered statistically significant.

DepMap portal (www.depmap.org) [55] browsing was used to correlate mRNA expression data in a cohort of breast cancer cell lines, expressed in Transcript Per Million (TPM), of *TPX2* versus SFK kinase genes, YAP/TAZ related genes, proliferative or CIN‐associated genes. Similarly, drug response data to dasatinib was retrieved in the format of Area Under the Curve (AUC), and correlated to mRNA expression data or the aneuploidy score as a CIN surrogate.

## 3 Results

### 3.1 Drug screening in *TPX2* ‐inducible expressing MDA‐MB‐453 cells identifies dasatinib as a selective hit

**Fig. 1:**
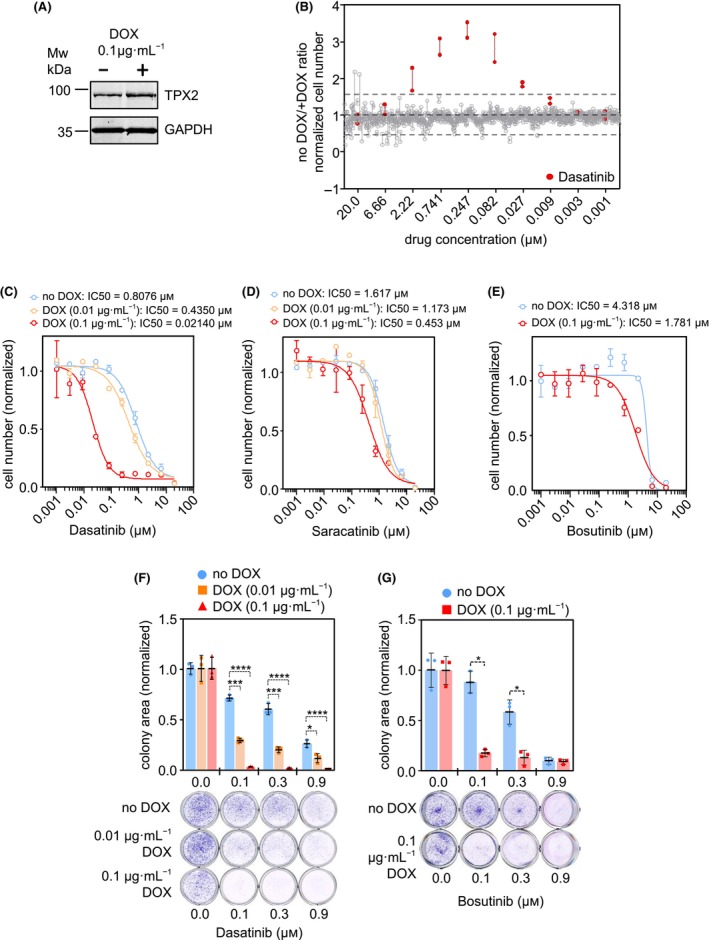
*TPX2* inducible overexpression leads to increased sensitivity to SRC kinase inhibitors. (A) MDA‐MB‐453 cells increase *TPX2* expression upon the inducible activation of the TET‐ON system by doxycycline (DOX). Representative blot of three experimental replicates. (B) Drug screening scatter plot of noDOX/+DOX ratios showing the response at each drug concentration tested in MDA‐MB‐453 cells during two cellular doubling rounds (6 days). Ratios for each compound (duplicates) are represented as gray dots, except dasatinib (highlighted in red). (C) 10‐point Concentration‐Response Curves (CRC) and IC50 calculation of dasatinib in MDA‐MB‐453 cells with two doses of DOX and drug incubation during 9 days. Mean cell number normalized vs. DMSO ± SEM (blue = noDOX control, orange = 0.01 μg·mL^−1^ DOX, red = 0.1 μg·mL^−1^ DOX). Representative graph of two experimental replicates. (D) 10‐point CRC and IC50 calculation of saracatinib in MDA‐MB‐453 cells with two doses of DOX and drug incubation during 9 days. Mean cell number normalized vs. DMSO ± SEM (blue = noDOX control, orange = 0.01 μg·mL^−1^ DOX, red = 0.1 μg·mL^−1^ DOX). Representative graph of two experimental replicates. (E) 10‐point CRC and IC50 calculation of bosutinib in MDA‐MB‐453 cells, with two doses of DOX and drug incubation during 9 days. Mean cell number normalized vs. DMSO ± SEM (blue = noDOX control, red = 0.1 μg·mL^−1^ DOX). Representative graph of two experimental replicates. (F) Colony formation assay, during 2 weeks, in MDA‐MB‐453 cells upon 0.1, 0.3, and 0.9 μm of Dasatinib treatment. The colony area is normalized vs. the DMSO‐treated cells ± SD. Two‐way ANOVA with Tukey multiple comparisons test: *P* < 0.0001 (****), *P* < 0.001 (***), *P* < 0.05 (*). (blue = noDOX control, orange = 0.01 μg·mL^−1^ DOX, red = 0.1 μg·mL^−1^ DOX). Representative graph of three experimental replicates. (G) Colony formation assay, during 2 weeks, in MDA‐MB‐453 cells upon 0.1, 0.3, and 0.9 μm bosutinib treatment. The colony area is normalized vs. the DMSO‐treated cells ± SD. Two‐way ANOVA with Tukey multiple comparisons test: *P* < 0.05 (*). (blue = noDOX control, red = 0.1 μg·mL^−1^ DOX). Representative graph of two experimental replicates.

We performed a drug sensitivity screening for mitotic and CIN‐related genes by creating a TET‐ON inducible gene expression system in the *HER2*‐positive breast cancer cell line MDA‐MB‐453 since this cell line is refractive to a large collection of drugs (Fig. S1A). Individual expression of nine different mitotic cDNAs (*BIRC5*, *CCNB1*, *CCNB2*, *ECT2*, *HEC1*, *MAD2*, *PRC1*, *PTTG1*, and *TPX2*) was verified by western blot after doxycycline (DOX) addition (Fig. S1B). The validated expressing cells were then subjected to the drug sensitivity screening using a drug panel of 60 small compounds (Table S1), in the absence (no overexpression of cDNA) or presence of DOX (overexpression of cDNA). Hence, we compare in the same genetic background, the differences in drug response upon overexpression of a particular gene of interest. The drug response data, after treatment with 10 concentration dilutions of each tested compound, was obtained by establishing a noDOX/+DOX proliferation ratio (Fig. S1C) and plotted to identify values above 1.5 indicating that +DOX induced a 50% decrease in cell proliferation. Out of the nine different cDNAs, inducible expression of *TPX2* (Fig. 1A) provided a strong differential response towards the SRC kinase inhibitor dasatinib (Fig. 1B).

To further validate this data, we performed Concentration‐Response Curves (CRC) to calculate the IC50 drug response of dasatinib (Fig. 1C) and other SRC kinase inhibitors such as saracatinib (Fig. 1D) and bosutinib (Fig. 1E). *TPX2* overexpression provides enhanced sensitivity to the three tested drugs in a dose‐dependent manner. Additionally, we tested the response to SRC inhibitors by performing cell colony formation assays in the presence of dasatinib or bosutinib (Fig. 1F,G), again demonstrating that *TPX2* overexpression increases the cellular sensitivity to SRC inhibitors. Interestingly, *TPX2* overexpression does not provide any differential response to imatinib (Fig. S2A), a specific inhibitor of the BCR‐ABL tyrosine‐kinase activity, which has very limited activity towards SRC kinase [56, 57], indicating that the mechanism associated with TPX2 overexpression might exclusively rely on the SFK kinase family and not on the ABL kinase activity. Similarly, we ruled out that the effect we observed was due to the action of dasatinib on other kinases such as c‐Kit, PDGR or EGFR, since we did not obtain a positive result in the initial drug panel screening with compounds specific for these kinases such as axitinib, imatinib, afatinib, lapatinib, dovitinib, crenolanib or dacomitinib.

**Fig. 2:**
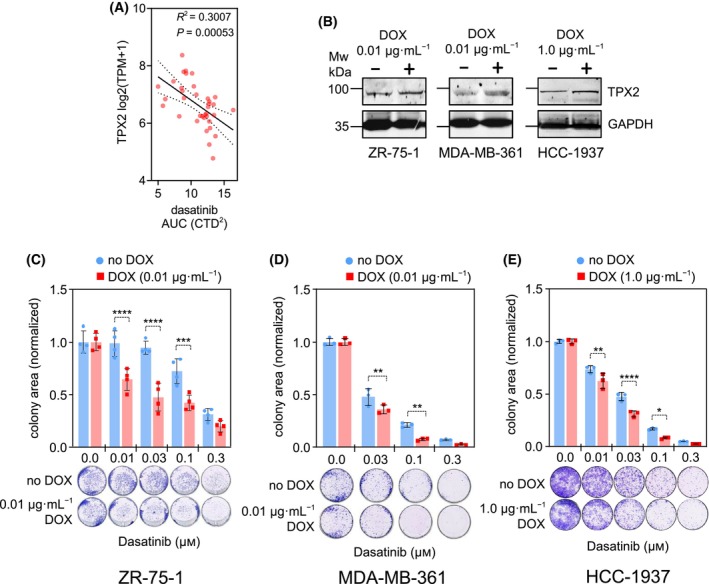
Dasatinib sensitivity upon *TPX2* overexpression in breast cancer cell lines. (A) DepMap portal retrieved data from 36 breast cancer cell lines, showing the correlation between *TPX2* expression (transcript per million – TPM) vs the sensitivity to dasatinib (area under the curve – AUC). The lower AUC, the more sensitivity to the drug. Correlation coefficient (*R*) was obtained by a Pearson correlation test. Statistical significance *P* < 0.05. (B) Inducible overexpression of *TPX2*, upon indicated doxycycline (DOX) concentrations, in the ZR‐75‐1, MDA‐MB‐361, and HCC‐1937 breast cancer cell lines. Representative graph of two experimental replicates. (C) Colony formation assay, during 2 weeks, in ZR‐75‐1 cells treated with the indicated concentrations of dasatinib. Representative graph of two experimental replicates. (D) Colony formation assay, during 2 weeks, in MDA‐MB‐361 cells treated with the indicated concentrations of dasatinib. Representative graph of two experimental replicates. (E) Colony formation assay, during 2 weeks, in HCC‐1937 cells treated with the indicated concentrations of dasatinib. Representative graph of two experimental replicates. The colony area is normalized vs. the DMSO‐treated cells ± SD. Two‐way ANOVA with Tukey multiple comparisons test: *P* < 0.0001 (****), *P* < 0.001 (***), *P* < 0.01 (**), *P* < 0.05 (*). (blue = noDOX control, red = DOX).

To verify that the effect on the response to dasatinib is common in other breast cancer cell lines, we first retrieved the *TPX2* mRNA expression data (transcripts per million –TPM) and dasatinib sensitivity values (area under the curve – AUC), from the DepMap portal, observing a strong significant correlation in a cohort of 36 breast cancer cell lines (Fig. 2A). Similar correlation data was obtained in breast cancer cell lines from the Cancer Therapeutics Response Portal from the Broad Institute [57] (Fig. S1D). Interestingly, it seems that the correlation of *TPX2* mRNA expression and dasatinib sensitivity is specific of breast cancer lines, not having significant data when analyzing both features in other cancer cell lines using the DepMap portal (Table S4). In addition, we further experimentally validated our data in other breast cancer cell lines such as ZR‐75‐1 (*ER* ^+^/*HER2* ^−^ luminal‐A subtype), MDA‐MB‐361 (*ER* ^+^/*HER2* ^+^ basal‐like subtype), and HCC‐1937 (triple negative basal‐like subtype). In the three cases, upon *TPX2* overexpression by doxycycline induction (Fig. 2B), we observed different levels of increased sensitivity to dasatinib (Fig. 2C–E), confirming that *TPX2* overexpression leads to dasatinib sensitivity in different breast cancer cell lines.

Since *TPX2* overexpression is also a bona fide marker for CIN [6, 26, 58], we correlated dasatinib sensitivity versus the aneuploidy score (as a surrogate indicator for CIN) using the breast cancer cell dataset in the DepMap portal. Dasatinib sensitivity does not correlate with aneuploidy or other CIN‐related genes such as *PRC1* or *FOXM1* (Fig. S2B). Similarly, as *TPX2* is also considered a proliferative gene [58, 59], we correlated dasatinib AUC data versus the expression of genes closely related to proliferation such as *MKI67*, *PCNA*, and *MCM2* (Fig. S2C). Only *MKI67* shows a correlation trend, but not significant, in the breast cancer cell line cohort. *PCNA* and *MCM2* show no correlation with dasatinib AUC indicating that cell proliferation index is not a determinant for dasatinib inhibitory effect.

### 3.2 SRC kinase signaling pathway evaluation upon TPX2 induction

**Fig. 3:**
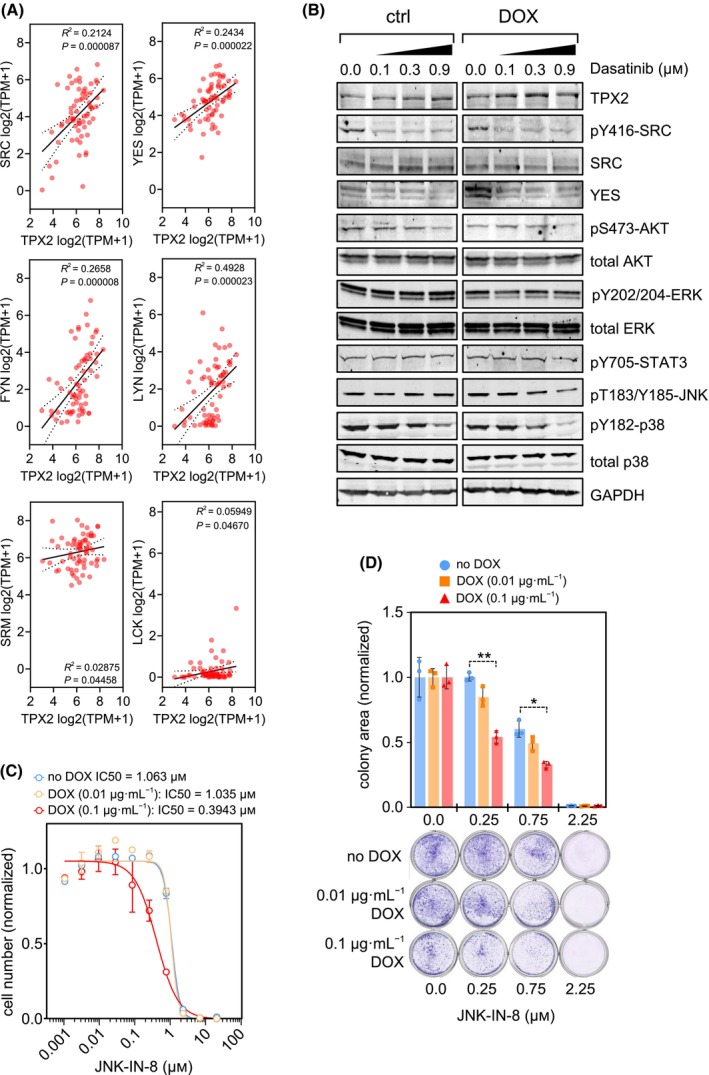
Evaluation of SFK downstream signaling upon *TPX2* inducible overexpression. (A) Correlation analysis of *TPX2* and SFK kinases genes (*SRC*, *YES*, *FYN*, *LYN*, *SRM*, and *LCK*) expression levels, using breast cancer cell lines data retrieved from the DepMap portal. Correlation coefficient (*R*) was obtained by a Pearson correlation test. Statistical significance *P* < 0.05. (B) Downstream SRC signaling analysis by western blot of MDA‐MB‐453 cells expressing *TPX2* (DOX) or control cells (ctrl), and treated with 0.1, 0.3, and 0.9 μm of dasatinib. GAPDH expression levels are used as a loading control. Representative graph of three experimental replicates. (C) 10‐point CRC and IC50 calculation of the JNK kinase inhibitor JNK‐IN‐8 in MDA‐MB‐453 cells, expressing TPX2 with two doses of DOX, and drug incubation during 9 days. Mean cell number normalized vs. DMSO ± SEM (blue = noDOX control, orange = 0.01 μg·mL^−1^ DOX, red = 0.1 μg·mL^−1^ DOX). Representative graph of two experimental replicates. (D) Colony formation assay, during 2 weeks, in MDA‐MB‐453 cells upon 0.1, 0.3 and 0.9 μm of JNK‐IN‐8 treatment. The colony area is normalized vs. the DMSO‐treated cells ± SD. Two‐way ANOVA with Tukey multiple comparisons test: *P* < 0.01 (**), *P* < 0.05 (*). (blue = noDOX control, orange = 0.01 μg·mL^−1^ DOX, red = 0.1 μg·mL^−1^ DOX). Representative graph of two experimental replicates.

To understand the increased sensitivity to dasatinib in *TPX2*‐overexpressing breast cancer cells, we first checked if the expression of SFK kinase genes correlates with *TPX2* expression levels. *TPX2* has a strong expression correlation with *SRC*, *YES*, *FYN*, and *LYN1* (Fig. 3A), which are SFKs known to exert a prominent role in breast cancer cell lines [60]. Of note, *TPX2* expression does not correlate with all SFK gene expression, such as *LCK*, most probably because there is very low *LCK* expression in breast cancer cell lines which is consistent with its lymphocyte specificity expression [61], or *SRM* kinase gene.

We then tested the expression and activation levels of SFKs (Fig. 3B). There are no significant changes in the SRC protein levels, upon *TPX2* overexpression, but a strong increase in YES kinase levels that drops down upon dasatinib addition. This reduction of YES protein levels, upon dasatinib addition, is concomitant with other reports using renal cancer cell lines [46]. Yet, we do not detect any activation of SRC signaling by testing the pTyr416 residue levels [62].

We also tested some major SRC downstream signaling pathways such as PI3K/AKT [63, 64, 65] and RAS/MEK/ERK [66, 67, 68] (Fig. 3B). Despite there being a description of a TPX2 and PI3K axis connection [69], we do not observe a significant alteration in PI3K signaling, since dasatinib treatment reduces AKT pSer473 levels equally in control and *TPX2*‐overexpressing cells. Concomitantly, when *TPX2*‐overexpressing cells are subjected to inhibition of PI3K (BYL‐719) or AKT (AZD‐5363), there are no changes in cell growth (Fig. S3A,B). As with RAS/MEK/ERK signaling, *TPX2* overexpression does not significantly alter the activation of ERK signaling (pTyr202/Thr204) (Fig. 3B). Interestingly, there is a differential response to RAS/MEK/ERK inhibitors such as dabrafenib (BRAF) or trametinib (MEK) (Fig. S3C,D). A possible explanation for this increased response to RAS/MEK/ERK inhibitors is that CIN cells seem to rely on the RAS/MEK/ERK axis to cope with the aneuploidy‐induced cellular stress [70]. SRC kinases also activate the STAT signaling pathway [71, 72], but we do not detect any alteration in STAT3‐pTyr705 phosphorylation upon *TPX2* overexpression (Fig. 3B). The stress signaling kinases p38 and JNK are also known downstream effectors of SRC activity [73, 74, 75, 76]. We do not observe any alteration in p38 activation (pTyr182) upon *TPX2* induction, and dasatinib suppresses p38 activation equally in control and *TPX2*‐overexpressing cells. On the contrary, we observe a differential response in the activation of JNK kinase (Fig. 3B). Whereas dasatinib has no effect on JNK‐Thr183/Tyr185 phosphorylation levels in parental cells, it leads to a reduction in JNK activation when *TPX2* is overexpressed. To further verify the possible impact of JNK in the TPX2‐mediated signaling, we tested cell survival upon JNK inhibition (Fig. 3C,D), and observed that *TPX2*‐overexpressing cells are more sensitive to the inhibitor JNK‐IN‐8 than control cells, indicating that JNK might participate in the dasatinib‐acquired sensitivity upon *TPX2* overexpression.

### 3.3 TPX2 overexpression leads to elevated YAP/TAZ signaling activity that is reverted by dasatinib addition

**Fig. 4:**
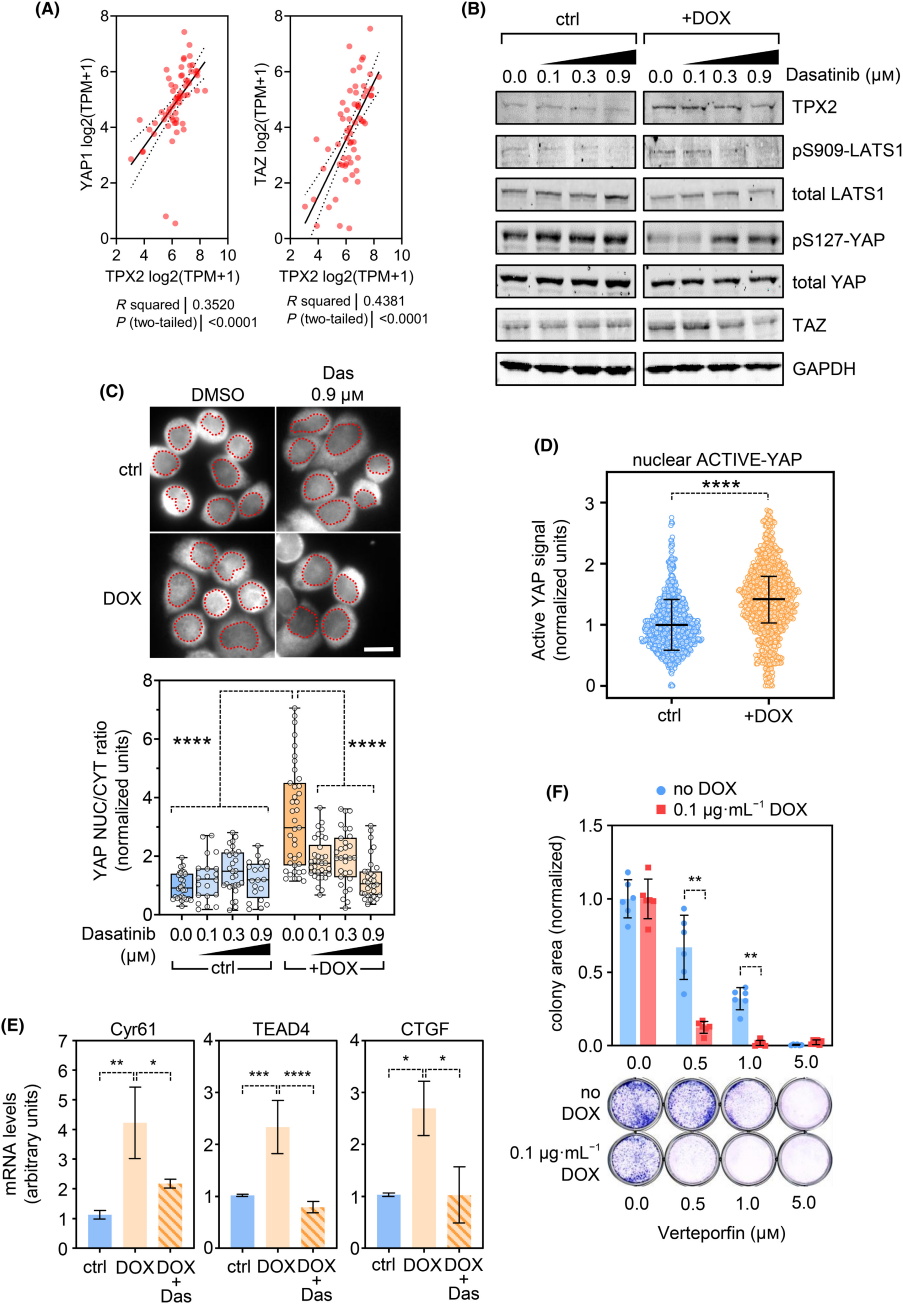
*TPX2* overexpression leads to increased YAP signaling. (A) Correlation analysis of *TPX2* expression levels of and the *YAP1* and *TAZ* transcription factors, using breast cancer cell lines data retrieved from the DepMap portal. Correlation coefficient (*R*) was obtained by a Pearson correlation test. Statistical significance *P* < 0.05. (B) Hippo/YAP signaling analysis by western blot of MDA‐MB‐453 cells expressing *TPX2* (DOX) or control cells (ctrl), treated with 0.1, 0.3, and 0.9 μm of Dasatinib. GAPDH expression levels are used as a loading control. Representative graph of three experimental replicates. (C) YAP nuclear/cytoplasm ratio analysis in MDA‐MB‐453 cells upon *TPX2* overexpression and dasatinib treatment. The red dotted line depicts the cell nucleus. Normalized data to the control untreated cells is represented in the histogram showing control cells (blue bars – ctrl) or *TPX2* expressing cells (orange bars – DOX) at 0.1, 0.3 and 0.9 μm of Dasatinib (light colored bars). Each dot represents a single microscopy field. One‐way ANOVA with Tukey multiple comparisons test: *P* < 0.001 (****). Representative graph of three experimental replicates. Scale Bar = 10 μm. (D) Immunofluorescence of active‐YAP in MDA‐MB‐453 cells upon *TPX2* expression with DOX. The nuclear signal is quantified with imagej software, and normalized vs. control data is plotted ± SD. Unpaired *T*‐Test analysis: *P* < 0.0001 (****). Representative graph of two experimental replicates. (E) RT‐qPCR gene expression test of *Cyr61*, *CGTF*, and *TEAD4* as surrogate markers of YAP transcription activity, showing mean mRNA levels (arbitrary units) ± SD. One‐way ANOVA with Tukey's multiple comparisons *post hoc* test *P* < 0.05 (*); *P* < 0.01 (**), *P* < 0.001 (***), *P* < 0.0001 (****). Representative graph of two experimental replicates. (F) Colony formation assay, during 2 weeks, in MDA‐MB‐453 cells upon 0.5, 1.0, and 5.0 μm of verteporfin treatment. The colony area is normalized vs. the DMSO‐treated cells ± SD. Two‐way ANOVA with Tukey multiple comparisons test: *P* < 0.01 (**). (blue = noDOX control, red = 0.1 μg·mL^−1^ DOX). Representative graph of two experimental replicates.

SFK kinases are important drivers of YAP/TAZ activity in several cancer types leading to tumor growth and metastasis [47, 77], and dasatinib efficiently inhibits the YAP/TAZ transcriptional survival signaling [40, 41, 46, 47, 78, 79]. Moreover, a proposed mechanism for YAP activation by SRC is mediated by the stress kinase JNK [46]. Since we observed an increase in YES kinase expression and differential response to JNK inhibitors, upon *TPX2* overexpression, we focused on the possibility of a YAP/TAZ signaling modulation. We first confirmed that *YAP* and *TAZ* genetic expression levels strongly correlate with *TPX2* expression in breast cancer cell lines (Fig. 4A).

*TPX2* overexpression leads to a reduction in the YAP inhibitory phosphorylation at Ser127, elevated levels of TAZ, and increased pSer909‐LATS1 (Fig. 4B), which is in accordance with the described YAP‐LATS1 feedback loop [80], and with previous data showing that TPX2 also leads to YAP/TAZ stabilization and activation [81, 82]. This YAP activation is reversed when dasatinib is added to *TPX2*‐overexpressing cells, restoring YAP‐Ser127 phosphorylation and recovering basal levels of TAZ protein (Fig. 4B). Noteworthy, we also observed the YAP activation (by reduction of pSer127‐YAP levels) in the triple negative HCC‐1937 cancer cell line (Fig. S4A). Concomitantly, *TPX2* induction promotes YAP nuclear shuttling in MDA‐MB‐453 cells, which is reversed by dasatinib (Fig. 4C). This data coincides with elevated levels of active‐YAP signal in the nucleus upon *TPX2* induction (Fig. 4D). We confirmed the *TPX2*‐dependent YAP/TAZ activation with increased expression of the surrogate genes *Cyr61*, *TEAD4*, and *CTGF* and its suppression by the addition of dasatinib (Fig. 4E). The DepMap portal also shows that *TPX2* expression levels in breast cancer cell lines strongly correlate with the expression of YAP/TAZ surrogate genes such as *CTGF*, *Cyr61*, *ANKRD1*, or *CRIM1* (Fig. S4B). Finally, the YAP/TAZ signaling inhibitor verteporfin [39] significantly inhibits the growth of *TPX2*‐overexpressing cells compared to control cells, demonstrating that *TPX2* overexpression leads to a YAP‐dependent growth (Fig. 4F).

In summary, our data show that *TPX2* leads to activation of the YAP/TAZ survival pathway, which confers enhanced sensitivity to dasatinib.

### 3.4 Dasatinib leads to a mitotic arrest in *TPX2* ‐overexpressing cells

Changes in *TPX2* expression, either down or upregulation, lead to mitotic aberrations that prevent proper chromosome segregation, leading to CIN [27, 28]. On the other hand, although SFKs allow progression through the early phases of the cell cycle [23] and dasatinib efficiently arrests the cell cycle in G_0_/G_1_ [83, 84, 85, 86], in recent years there is strong evidence that SFKs are also functional during mitosis [67, 87, 88, 89, 90, 91].

**Fig. 5:**
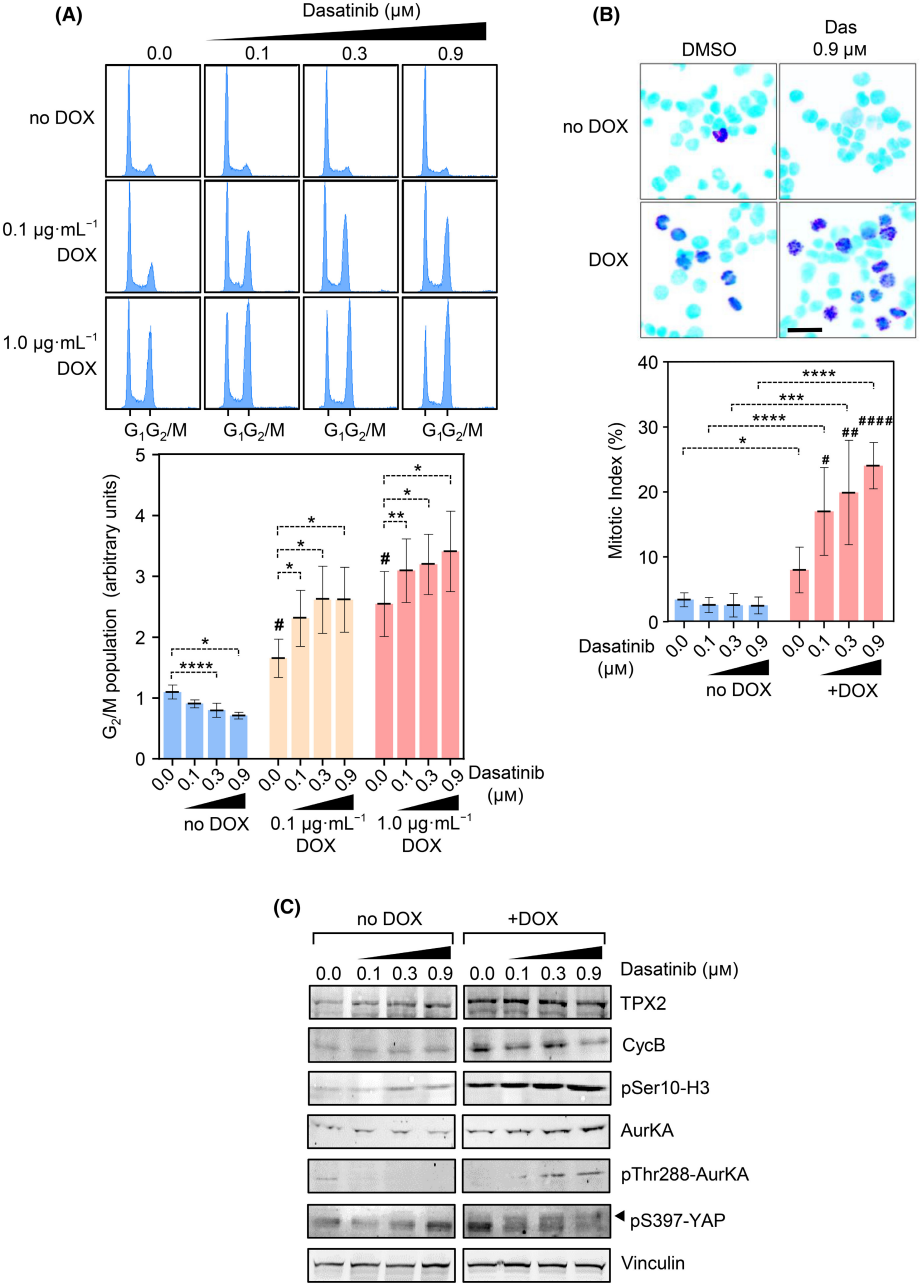
Dasatinib strengthens the mitotic arrest mediated by *TPX2* overexpression. (A) Cell cycle profiling by DAPI DNA staining and flow cytometry analysis of MDA‐MB‐453 cells. Cells incubated with DOX (0.01 and 0.1 μg·mL^−1^) for TPX2 expression, were also treated with 0.1, 0.3 and 0.9 μm of Dasatinib. The G_1_ and G_2_/M peaks are indicated at the bottom of the cell cycle profiles, and the quantification of the G_2_/M percentage of cells (±SD) is shown in the bottom histogram. Two‐way ANOVA with Tukey multiple comparisons test comparing dasatinib impact: *P* < 0.0001 (****) *P* < 0.01 (**), *P* < 0.05 (*); or TPX2 expression impact versus no DOX treated cells: *P* < 0.05 (#). (blue = no DOX control, orange = 0.1 μg·mL^−1^ DOX, red = 1.0 μg·mL^−1^ DOX). Representative graph of three experimental replicates. (B) Mitotic index quantification by phospho‐Ser10 Histone H3 (pH3) immunofluorescence upon DOX incubation and dasatinib treatment. The upper panel shows a representative image of MDA‐MB.453 cells stained with DAPI for DNA (light blue) and pH3 depicting mitotic cells (dark purple). The bottom histogram shows the quantification (±SD) at different concentrations of dasatinib. Two‐way ANOVA with Tukey multiple comparisons test, comparing *TPX2* expression impact: *P* < 0.0001 (****) *P* < 0.001 (***), *P* < 0.05 (*); or dasatinib impact with in the TPX2 expressing cohort; *P* < 0.0001 (####) *P* < 0.01 (##) *P* < 0.05 (#). (blue = no DOX control, red = 1.0 μg·mL^−1^ DOX). Representative graph of three experimental replicates. Scale Bar = 25 μm. (C) Biochemical analysis by western blot of mitotic markers activation, and YAP‐Ser397 phosphorylation, of MDA‐MB‐453 cells expressing *TPX2* (DOX) or control cells (ctrl), treated with 0.1, 0.3, and 0.9 μm of Dasatinib. GAPDH expression levels are used as a loading control. Representative graph of three experimental replicates.

We thus evaluated the cell cycle status upon *TPX2* overexpression and dasatinib intervention in MDA‐MB‐453 cells. As expected, dasatinib addition to uninduced cells leads to a significant reduction in the G_2_/M proportions, whereas *TPX2* induction leads to a G_2_/M cell cycle arrest (Fig. 5A). Interestingly, when *TPX2*‐overexpressing cells are treated with dasatinib, there is a strong enhancement of the G_2_/M phase arrest in a dasatinib and *TPX2* dose‐dependent manner (Fig. 5A). This cell cycle arrest is mostly due to a mitotic arrest, as depicted by pSer10‐H3 staining and quantification (Fig. 5B,C). The mitotic arrest led by dasatinib is corroborated by increased expression of mitotic proteins such as Cyclin B (CycB) or Aurora kinase A (AurKA), and also a notable increase in phospho‐AurKA signal at the activatory residue Thr288 (Fig. 5C). In parallel, we also observed an equivalent G_2_/M arrest, upon dasatinib addition, in cells previously treated with low levels of nocodazole to mimic the *TPX2*‐mediated arrest (Fig. S4C). This indicates that cells arrested in mitosis become sensitive to dasatinib.

It is well known that a mitotic arrest influences the Hippo‐YAP/TAZ signaling in different ways. On one hand, the mitotic kinase CDK1 phosphorylates and activates YAP during the G_2_/M phase [92, 93]. On the other hand, CDK1 phosphorylates and inactivates TAZ during mitosis [94], and also phosphorylates and activates LATS kinases upon mitotic stress [95, 96]. Other mitotic kinases such as AurKA can modulate the Hippo‐YAP/TAZ axis signaling by phosphorylating YAP‐Ser397 [97]. Noteworthy, phosphorylation at YAP‐Ser397 is controversial in terms of activity, as it plays as a YAP inhibitory event leading to YAP degradation [98], but also as a YAP activator mechanism, precisely upon *TPX2* overexpression and AurKA phosphorylation in breast cancer cell lines [97].

We, therefore, evaluated the levels of YAP‐Ser397 phosphorylation, detecting an increased level in YAP‐Ser397 phosphoresidue when *TPX2* overexpressing cells are cultured under dasatinib, whereas control cells do not alter YAP‐Ser397 phosphorylation levels (Fig. 5C). This YAP‐Ser397 phosphorylation is coincident with the recovery of the inhibitory YAP‐Ser127 signal and reduction of the active YAP signal (Fig. 4B), and the cytoplasmic retention (Fig. 4C), suggesting that in this context YAP‐Ser397 reflects inhibition of YAP activity.

Overall, dasatinib leads to an efficient cell cycle arrest in mitosis in *TPX2*‐overexpressing cells, leading to YAP inactivation by phosphorylation at Ser397 and Ser127 residues.

### 3.5 *TPX2* expression and *YAP* activation correlation analysis in breast cancer patient‐derived samples

**Fig. 6:**
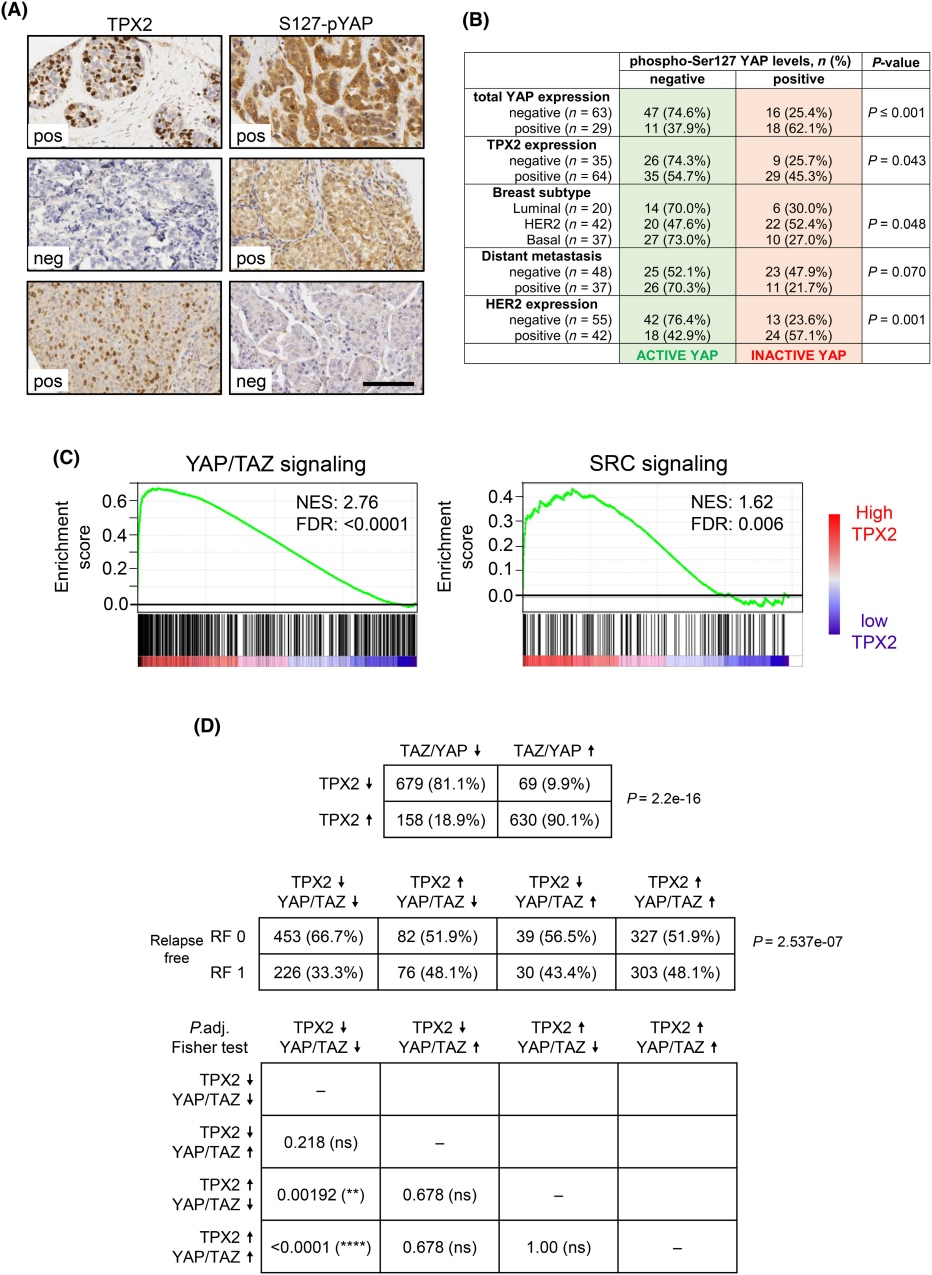
Correlation analysis of *TPX2* expression and YAP activation of a breast cancer tumoral microarray. (A) Immunohistochemistry of TPX2 and pS127‐YAP in 99 grade‐3 breast cancer paraffin‐embedded samples. The upper row shows an example of breast carcinoma with a positive signal for both biomarkers. The middle row is an example of breast carcinoma with no TPX2 expression and with S127‐pYAP positive signal (indicative of YAP inactivation). The bottom panel indicates an example of breast carcinoma with positive expression of TPX2 and negative S127‐pYAP expression (indicative of YAP activation). Pictures were obtained at 40× magnification. Scale Bar = 100 μm. (B) Quantification and statistical analysis table of 99 samples stained as in panel (A) showing the relationship between pS127‐YAP signal and other immunohistochemical features, such as expression of YAP, TPX2 and HER2, metastasis capacity, and tumoral subtypes. (C) Gene Set Enrichment Analysis (GSEA) of breast invasive ductal carcinoma samples from the METABRIC project, accordingly to *TPX2* expression levels, showing enhanced YAP/TAZ signaling [53] and SRC signaling (SRC_UP.V1_DN) [111]. Normalized Enrichment Score (NES) and FDR qValue are indicated in each plot. (D) Correlation analysis of *TPX2* and the YAP/TAZ signature expression (z‐score) in breast cancer samples from the METABRIC project, evaluating the influence on Relapse Free survival (RF0 = no relapse; RF1 = positive relapse). Statistical analysis by adjusted Fisher test: *P* < 0.0001 (****); *P* < 0.01 (**).

To evaluate the clinical translation of our findings, we examined whether *TPX2* expression levels and *YAP* activation (by YAP‐pSer127 inhibitory phosphorylation) are coincident biomarkers in an array of 99 invasive ductal breast carcinoma samples (Fig. 6A). We first tested that YAP‐pSer127 staining inversely correlates with total YAP levels (not shown), thus validating the YAP‐pSer127 labeling. Although a fraction of samples show low YAP‐pSer127 and no *TPX2* expression, the majority (54%) of the *TPX2*‐positive cases also have increased YAP activity (Fig. 6B). We also compared other clinical features such as distant metastasis and subtype classification. 70% of patients with distant metastasis have negative YAP‐pSer127 staining, indicating that YAP/TAZ activation is also a hallmark of aggressiveness. Since MDA‐MB‐453 is a Her2‐positive cell line, we also evaluated the *HER2* status in the analyzed tumors, showing most cases being negative for YAP‐pSer127 staining.

To reinforce this data, we explored the METABRIC Project database [51] by performing a Gene Set Enrichment Analysis (GSEA) of 1536 breast invasive ductal carcinoma samples according to the *TPX2* expression levels. *TPX2* high expression correlates with an increase in YAP/TAZ and SRC‐dependent signaling (Fig. 6C), and we obtained similar data using the TCGA database (Fig. S4D). The METABRIC dataset indicates that the expression of *TPX2* and the YAP/TAZ signature strongly correlate (Fig. 6D), and has a significant impact on malignancy as depicted by Relapse Free (RF) survival. The few samples where *TPX2* and YAP/TAZ signatures do not correlate show no differences in RF, thus suggesting that *TPX2* overexpression needs an elevated YAP/TAZ signaling to provide a poor tumoral prognosis. In summary, breast cancer samples that show high levels of *TPX2* often present enhanced YAP/TAZ activation, making dasatinib a therapeutic option, especially in the more aggressive tumors.

## 4 Discussion

Chromosomal Instability is a hallmark of cancer, being a feature not present in healthy primary cells. Therefore, it might be a bona fide biomarker for cancer therapy, and strong efforts have been done trying to find drugs that selectively kill cancer cells based on their CIN rate [9, 10, 99]. Despite some of these studies show positive correlation between CIN and certain therapies [99], the final results generally demonstrated that CIN is difficult to target, and the few drugs depicted are very toxic compounds [10]. In addition, high‐CIN cancer cells often show elevated proliferation rates, and this feature can be responsible for positive responses to drugs based on protein turnover or energy balance [9]. The fact that CIN cells are already very heterogeneous in their genetic dosage can explain why they do not respond to therapy uniquely based on the CIN rate. Moreover, this genetic plasticity provides adaptation to external insults, being an engine for therapy resistance [100, 101].

CIN is characterized by the expression of particular genetic signatures, mainly based on mitotic and cell cycle genes [6, 11, 12, 13, 14, 15, 16]. Using a gene‐inducible expression system, we screened cancer cells, individually expressing a collection of CIN‐related genes, against a selected library of drugs. This approach avoids the enormous differences between different cell lines, as we compare the same cell line expressing or not the gene of interest. Thus, the genetic background is the same, and we can depict the precise therapy dependency on one gene. We found that the expression of *TPX2*, a major representative gene in CIN signatures, provides strong sensitivity towards SRC kinase inhibitors such as dasatinib, saracatinib, and bosutinib (Fig. 1). Interestingly, none of the other tested genes provided such a response. This indicates that the drug response is *TPX2*‐specific rather than CIN‐associated. Indeed, there is no significant correlation between dasatinib response and CIN rate, or expression of other CIN‐related genes, according to the DepMap database (Fig. S2). Worth mentioning, other research groups have shown that SRC inhibitors can affect the proliferation of CIN cells, although not as a general mechanism. Firstly, Schukken et al. [102] made a similar screen approach by confronting a small panel of drugs in cells depleted for *MAD2* (leading to severe aneuploidy and CIN in the short term) and showed an increased sensitivity towards the SRC inhibitor SKI606 (bosutinib). Here, bosutinib exacerbates the mitotic aberrations generated upon *MAD2* depletion, by increasing the polymerization rates of microtubules, evidencing similarities to our cell cycle analysis showing how dasatinib leads to a strong mitotic arrest upon TPX2 overexpression (Fig. 5). Whether this alteration depends on microtubule dynamics, in the TPX2‐overexpressing cells, remains to be determined. Secondly, in an exhaustive *in silico* study using single‐nucleotide polymorphism (SNP) data from the TCGA, researchers identified 17 different CIN signatures aiming to predict drug response and new drug targets [103]. Interestingly, this study describes that dasatinib precisely correlates with a CIN signature (CX10), which originated from a defective non‐homologous end joining (NHEJ) DNA repairing mechanism. The TPX2/AurKA complex is implicated in DNA repair, as it interacts with essential DNA repair factors such as BRCA1/2, PARP1, and 53BP1. TPX2 is known to accumulate at DSBs where it negatively regulates 53BP1, thus modulating homologous recombination (HR), replication fork stability, and inhibiting NHEJ [104, 105, 106, 107].

The association between SRC and the Hippo‐YAP/TAZ pathway is well documented in the literature [44], and a tightly controlled SRC‐YAP signaling axis determines therapeutic response to dasatinib mediated by the stress kinase JNK [46]. Since we observed that *TPX2*‐overexpressing cells have increased YES kinase levels, and JNK kinase inhibition reduced their proliferation capacity (Fig. 3), we deeply analyzed the impact of *TPX2* overexpression on YAP/TAZ signaling, demonstrating that TPX2 high levels promote YAP activation and this is probably the reason for dasatinib‐increased sensitivity (Fig. 4). Our data is concomitant with previous works showing that *TPX2* overexpression increases YAP signaling in breast cancer cells, and this is dependent on Aurora A kinase activity [81, 82, 97]. Moreover, Aurora A is also known for modulating SRC through the cofactor NEDD9, affecting dasatinib response [108].

The reduced proliferation in TPX2‐overexpressing cells, upon dasatinib treatment, can be explained by the strong mitotic arrest observed (Fig. 5). Although SRC kinases are primarily implicated in mitogenic signaling, there is also evidence that they can modulate mitosis. SRC‐family kinases modulate microtubule polymerization rates [102, 109], a process where TPX2 also has an important role. Similarly, *v‐SRC* expression overrides the spindle assembly checkpoint leading to chromosome missegregation [91, 110]. The mitotic alterations generated by *TPX2* overexpression can synergize with the inhibition of SRC by dasatinib, leading to the observed mitotic arrest, and further studies need to be done to determine the precise mechanism of action.

Intending to translate our data to real cancer samples, we explored the breast cancer data from the METABRIC project, and a cohort of breast invasive ductal carcinoma samples (Fig. 6). The data obtained show that indeed elevated *TPX2* strongly correlates with increased SRC and YAP signaling and that TPX2 confers poor prognosis only when YAP/TAZ signaling is elevated. About 54% of *TPX2*‐positive breast tumors also harbor *YAP* activation, demonstrating that both biomarkers are present in a significant proportion of breast tumors. Interestingly, these tumors seem to be the most aggressive. Our experimental IHC data (Fig. 6A,B) differs in the percentage of tumors with *TPX2* overexpression and *YAP* activation, when compared to the *in silico* data from the METABRIC platform. We infer YAP activation in the METABRIC data by evaluating the YAP/TAZ expression signature. An explanation for this difference is the fact that the YAP/TAZ transcriptional signature is also bona fide indicative of cell proliferation, as it is also TPX2 expression. Collectively, our data suggest *TPX2* overexpression and YAP/TAZ signaling as putative biomarkers for alternative dasatinib therapy in aggressive breast cancer.

## 5 Conclusions

Overexpression of the CIN‐associated gene *TPX2* provides enhanced sensitivity to the SRC kinase inhibitor dasatinib in breast cancer cell lines. This increased dasatinib sensitivity is based on an elevated YAP/TAZ transcriptional activity, due to *TPX2* overexpression. *TPX2* elevated levels lead to a mitotic arrest that is strengthened by Dasatinib addition. Analysis in patient‐derived breast cancer samples shows a strong correlation between high *TPX2* expression levels and YAP/TAZ signaling activation, and this provides a poor prognosis.

## Conflict of interest

CM and MJL are employees and shareholders of Eli Lilly Company.

## Author contributions

CM performed the drug screening and *in vitro* experiments, with the help of BO, AM‐V, CB, MG, and AAV. NS‐G performed *in vitro* experiments and the METABRIC and TCGA bioinformatic analysis. AT and GM‐B performed the breast tumoral samples analysis. MJL provided intellectual input and supervised the drug screening. GdC designed and supervised the study. All authors participated in the data analysis, and GdC wrote the paper with the help of NS‐G, CM, and MJL.

### Peer review

The peer review history for this article is available at https://www.webofscience.com/api/gateway/wos/peer‐review/10.1002/1878‐0261.13602.

## Supporting information


**Fig. S1.** Drug Screen in MDA‐MB‐453 cells expressing CIN‐associated genes.
**Fig. S2.** Dasatinib response correlation to proliferation and aneuploidy genes markers.
**Fig. S3.** Response of TPX2‐expressing cells to PI3K/AKT and RAS/MEK/ER inhibitors.
**Fig. S4.** Correlation of *TPX2* expression and YAP/TAZ signaling markers.

**Table S1.** Screening drug collection.
**Table S2.** CIN‐associated cDNAs source, accession number, and PCR cloning oligos.
**Table S3.** List of antibodies used for western blot analysis.
**Table S4.** Correlation data of TPX2 expression and dasatinib sensitivity in different cancer type cell lines.

## Data Availability

The *in silico* data that support some of the findings of this study are generated by using the following tools: The Dependency Map (DepMap) portal at https://depmap.org/portal/, [http://doi.org/10.1016/j.cell.2017.06.010]. The Cancer Genome Atlas (TCGA) portal at https://www.cancer.gov/tcga. The Molecular Taxonomy of Breast Cancer International Consortium (METABRIC) at https://www.mercuriolab.umassmed.edu/metabric, [http://doi.org/10.1038/nature10983]. Any other data that support the findings of this study are available from the corresponding author [gdecarcer@iib.uam.es] upon reasonable request.
