# Supplementary material for: TPX2 overexpression promotes sensitivity to dasatinib in breast cancer by activating YAP transcriptional signaling
Source: Mol Oncol. 2024 Feb 15;18(6):1531–51. doi: 10.1002/1878-0261.13602 (PMC11161735; doi:10.1002/1878-0261.13602)
Supplement: Supplementary file 1 — Fig. S1. Drug Screen in MDA‐MB‐453 cells expressing CIN‐associated genes. Fig. S2. Dasatinib response correlation to proliferation and aneuploidy genes markers. Fig. S3. Response of TPX2‐expressing cells to PI3K/AKT and RAS/MEK/ER inhibitors. Fig. S4. Correlation of TPX2 expression and YAP/TAZ signaling markers. [file MOL2-18-1531-s002.zip › MOLONC-23-0828_Supplementary Figure legends_FINAL_20240124.docx]

Supplementary Figure 1: Drug Screen in MDA-MB-453 cells expressing CIN-associated genes.

**A).** Drug sensitivity data (Log10 IC50 value) of the parental (no DOX) MDA-MB-453 cell line across the panel of drugs used in the screen. The red arrow highlights dasatinib value.

**B).** Inducible expression test for the nine cDNAs in MDA-MB-453 cells upon doxycycline addition (1.0 μg/ml) for 24 hours.

**C).** Cartoon showing how the drug screen was performed in MDA-MB-453 cells. 10 serial dilutions of each drug are added to cells in duplicates, and plates are treated either with doxycycline media or with regular media for cDNA expression. Drug incubation lasted two doubling times, and DNA-stained cells were counted. The number of cells was normalized by the average cell number in the DMSO-treated cells (no drug), and then the ratio of no DOX/+ DOX was calculated for each drug concentration.

**D).** Z-scored Pearson correlation coefficients between small-molecule sensitivity data, expressed as areas under concentration-response curves (AUCs), with basal *TPX2* gene-expression measurements, expressed as log2 robust-multi-array-average values, in Breast Cancer cell lines, retrieved from the Cancer Therapeutics Response Portal (CTRP). (<https://portals.broadinstitute.org/ctrp.v2.1/?featureName=TPX2>)

Supplementary Figure 2: Dasatinib response correlation to proliferation and aneuploidy genes markers.

**A)** 10-point Concentration-Response Curves (CRC) and IC50 calculation of imatinib, in MDA-MB-453 cells expressing TPX2, and drug incubation during 9 days. Mean cell number normalized vs. DMSO +/- SEM (blue = no DOX control, red = 0.1 μg/ml DOX).

**B)** DepMap portal retrieved data from breast cancer cell lines, showing the correlation between the sensitivity to dasatinib (area under the curve - AUC) versus the aneuploidy score (based on the ABSOLUTE copy number data from the Cancer Cell Line Encyclopedia (*Ghandi, M., et al. Nature 2019. v569, 503–508. doi: 10.1038/s41586-019-1186-3*)), or the expression of the CIN related genes *PRC1* and *FOXM1* in transcripts per million (TPM).

**C)** Similar DepMap portal correlation analysis of dasatinib sensitivity (AUC) versus the expression levels (TPM) of the proliferating genes *MKI67, PCNA* or *MCM2*

Supplementary Figure 3: Response of *TPX2*-expressing cells to PI3K/AKT and RAS/MEK/ER inhibitors.

**A)** PI3K kinase inhibitor BYL-719.

**B)** AKT inhibitor AZD-5363.

**C)** RAF inhibitor dabrafenib.

**D)** MEK inhibitor trametinib.

**Left Panels:** 10-point CRC and IC50 calculation of the indicated drug in MDA-MB-453 cells expressing TPX2, during 3 doubling times. Mean cell number normalized vs. DMSO +/- SEM (blue = no DOX control, orange = 0.01 μg/ml DOX, red = 0.1 μg/ml DOX).

**Middle panels:** Colony formation assay, during two weeks in MDA-MB-453 cells upon treatment with the indicated inhibitors. The colony area is normalized vs. the DMSO-treated cells +/- SD. Two-way ANOVA with Tuckey multiple comparisons test: p<0.01 (**), p<0.05 (*). (blue = no DOX control, orange = 0.01 μg/ml DOX, red = 0.1 μg/ml DOX).

**Right panels:** representative image of the colony formation assay.

Supplementary Figure 4: Correlation of TPX2 expression and YAP/TAZ signaling markers.

**A).** YAP signaling analysis by western blot in HCC-1937 cells overexpressing *TPX2* (DOX 1.0 μg/ml) or control cells (ctrl), treated with 0.01 μM, 0.03 μM, and 0.1 μM of Dasatinib. GAPDH expression levels are used as a loading control.

**B).** Correlation analysis of *TPX2* and YAP/TAZ signaling surrogate genes (*Cyr61*, *CTGF*, *ANKRD1*, and *CRIM1*) expression levels in transcripts per million (TPM), using breast cancer cell lines data retrieved from the DepMap portal.

**C).** Cell cycle profiling, by DAPI DNA staining and flow cytometry analysis, of MDA-MB-453 cells treated with 20 nM nocodazole for 24 hours, and then 0.9 μM dasatinib for another 24 hours. G_1_ and G_2_/M peak percentages are indicated inside the plots.

**D).** Gene Set Enrichment Analysis (GSEA) of 1082 patients breast invasive ductal carcinoma samples from the TCGA-PanCanAtlas and BRCA project (*Cancer Genome Atlas Research. Nat Genet. 2013. v45(10):1113-20. doi: 10.1038/ng.2764*), accordingly to *TPX2* expression levels, showing enhanced YAP/TAZ signaling. (Cordenonsi_YAP_Conserved_Signature) (*Cordenonsi et al., Cell. 2011 v147(4):759-72. doi: 10.1016/j.cell.2011.09.048)* and SRC signaling (SRC_UP.V1_DN) (*Bild. A.H, et al., Nature. 2006. v439(7074):353-7. doi: 10.1038/nature04296*). Patients were classified according to *TPX2* expression with a membership probability estimated by bootstrap (*Bueno-Fortes S, et al., Bioinform Adv. 2023. v3(1). doi: 10.1093/bioadv/vbad037*). Gene Set Enrichment Analysis (GSEA) was performed by comparing high versus low *TPX2* expressing samples. The top 25 terms of C6: Oncogenic Signature were analyzed. FDR q<0.05 was considered significant.
