# Supplementary figures and images for: TPX2 overexpression promotes sensitivity to dasatinib in breast cancer by activating YAP transcriptional signaling
Source: Mol Oncol. 2024 Feb 15;18(6):1531–51. doi: 10.1002/1878-0261.13602 (PMC11161735; doi:10.1002/1878-0261.13602)

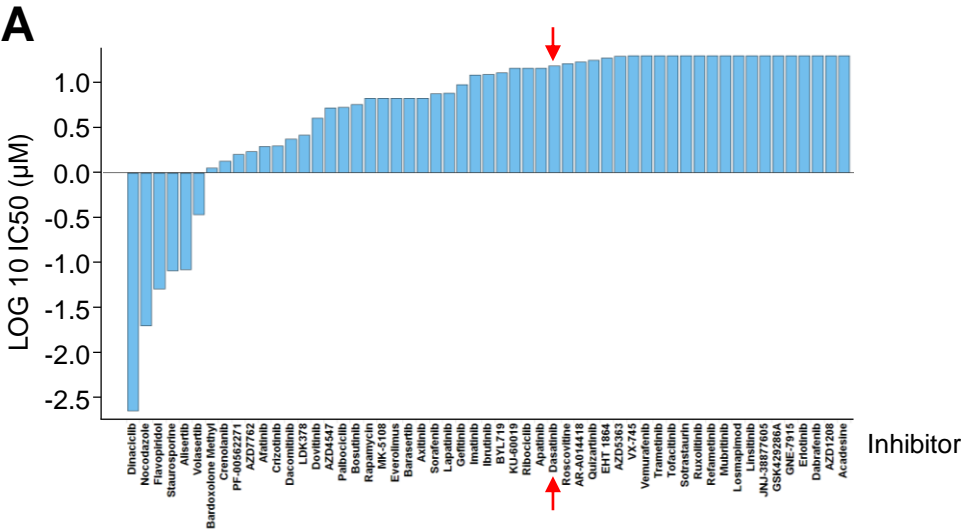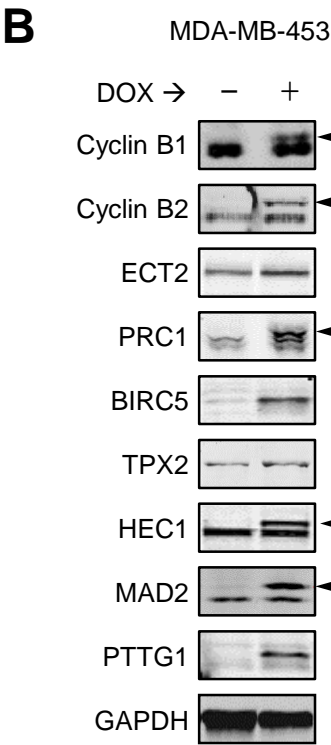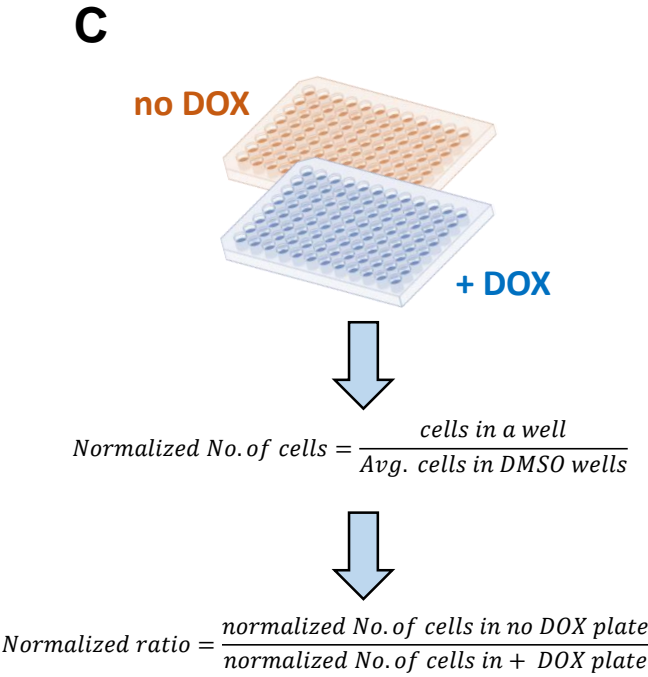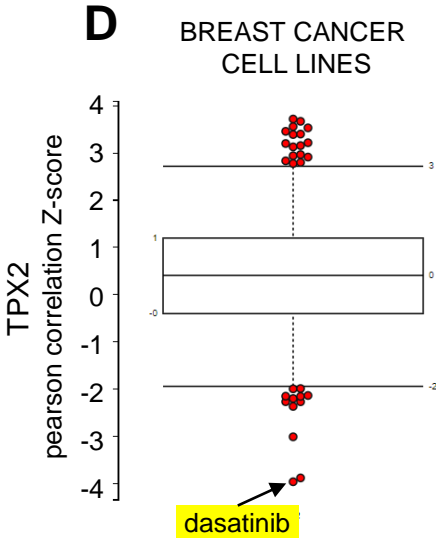

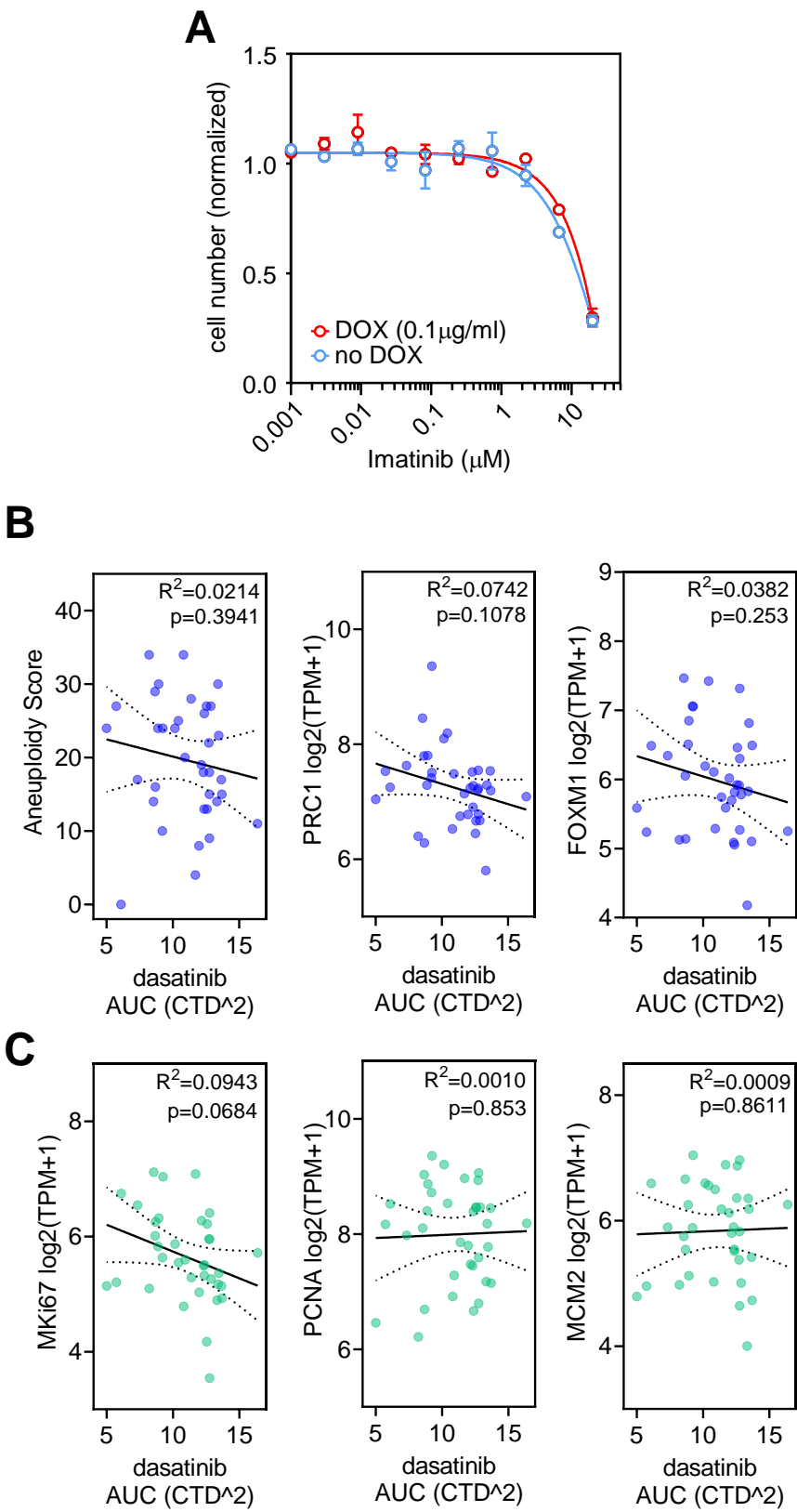

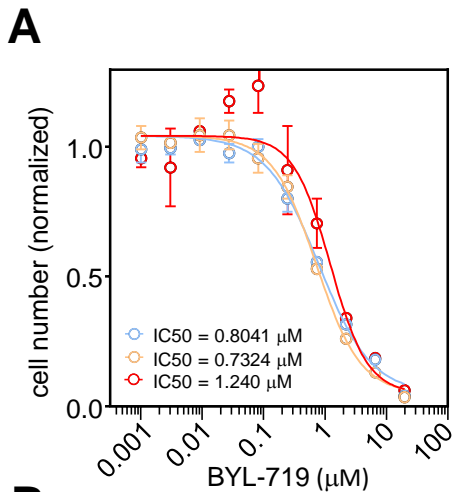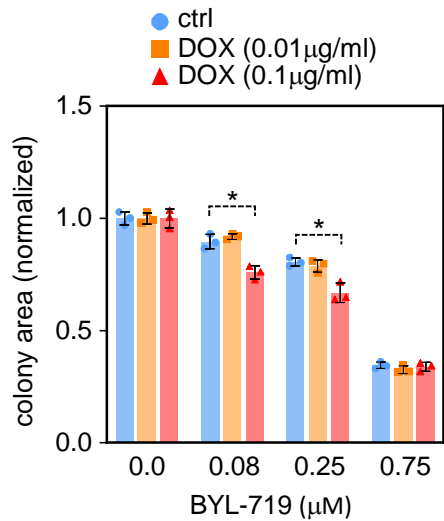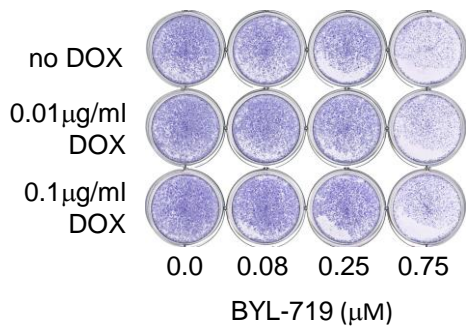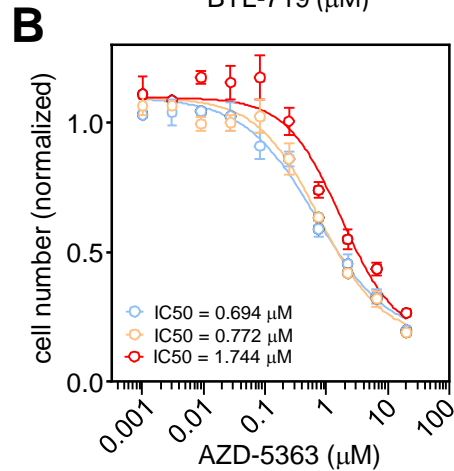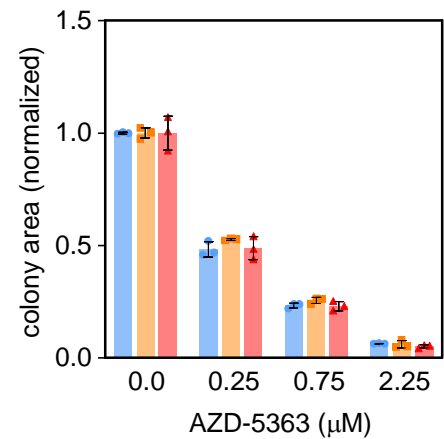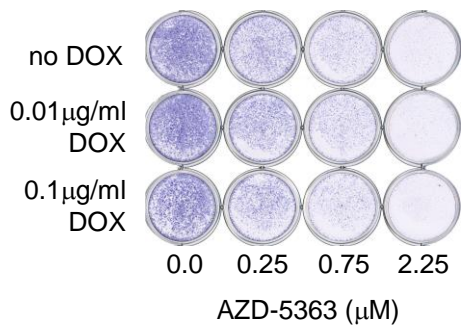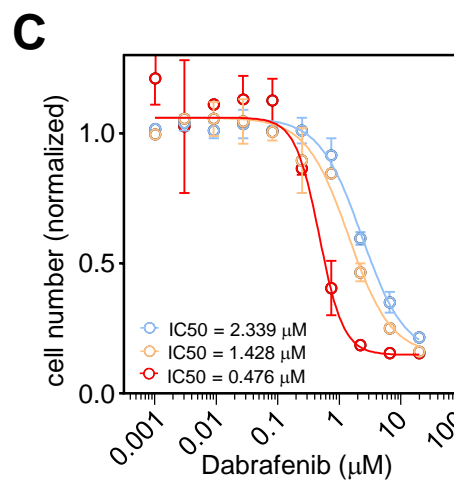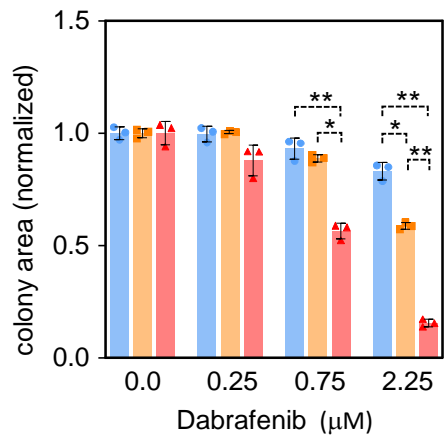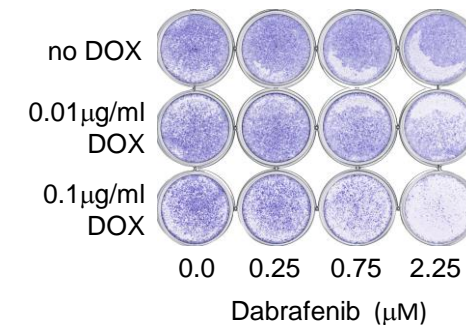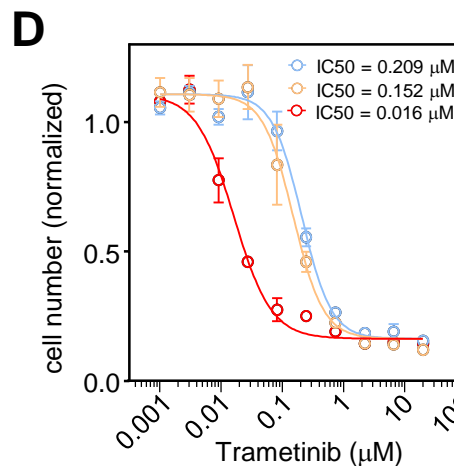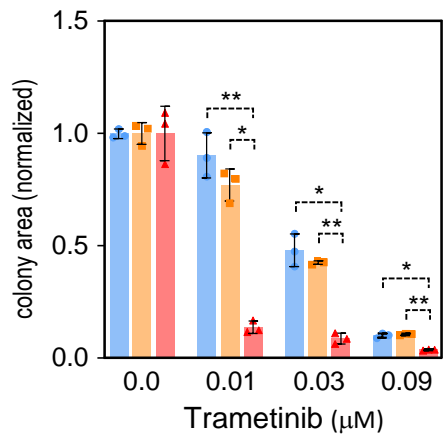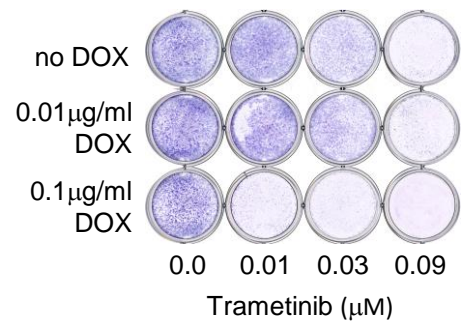

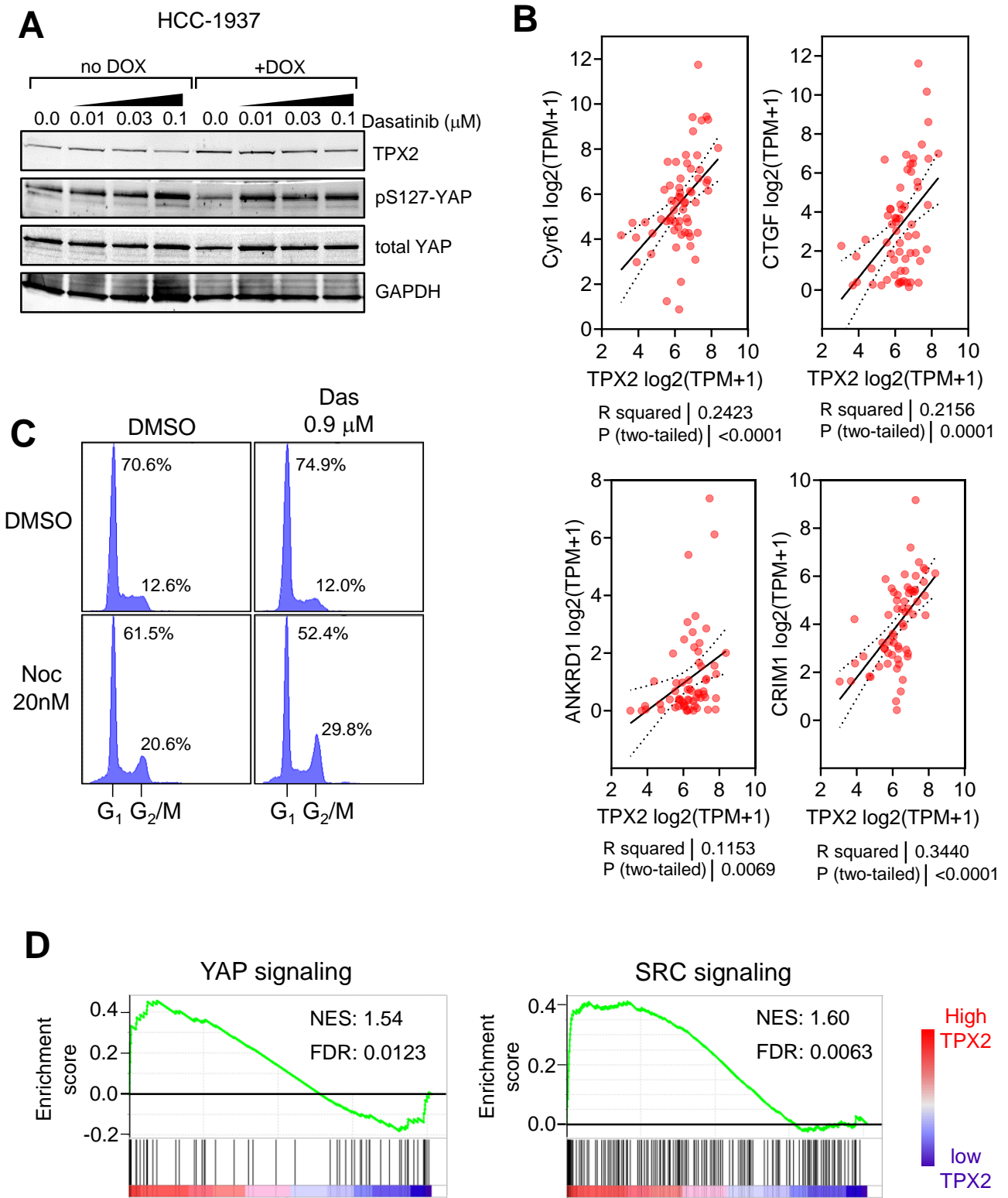

Supplement: Supplementary file 1 — Fig. S1. Drug Screen in MDA‐MB‐453 cells expressing CIN‐associated genes. Fig. S2. Dasatinib response correlation to proliferation and aneuploidy genes markers. Fig. S3. Response of TPX2‐expressing cells to PI3K/AKT and RAS/MEK/ER inhibitors. Fig. S4. Correlation of TPX2 expression and YAP/TAZ signaling markers. [file MOL2-18-1531-s002.zip › MOLONC-23-0828_supplementary figures_FINAL_20240124.pdf]
