# Supplementary material for: TPX2 overexpression promotes sensitivity to dasatinib in breast cancer by activating YAP transcriptional signaling
Source: Mol Oncol. 2024 Feb 15;18(6):1531–51. doi: 10.1002/1878-0261.13602 (PMC11161735; doi:10.1002/1878-0261.13602)
Supplement: Supplementary file 2 — Table S1. Screening drug collection. Table S2. CIN‐associated cDNAs source, accession number, and PCR cloning oligos. Table S3. List of antibodies used for western blot analysis. Table S4. Correlation data of TPX2 expression and dasatinib sensitivity in different cancer type cell lines. [file MOL2-18-1531-s001.docx]

Marugan et al. (Suplementary tables)

**Supp. Table 1. Screening drug collection.**

The table summarizes the collection of 60 small compounds used in the screening, by name, target molecule, and the clinical status (cpd: compound).

| **#** | **Inhibitor** | **Target** | **Status** |
| --- | --- | --- | --- |
| 1 | Afatinib | EGFR, HER2 | FDA approval |
| 2 | Axitinib | c-Kit, VEGFR, PDGFR | FDA approval |
| 3 | Bosutinib | SRC | FDA approval |
| 4 | Crizotinib | c-Met, ALK | FDA approval |
| 5 | Dabrafenib | Raf | FDA approval |
| 6 | Dasatinib | BCR-ABL, SRC | FDA approval |
| 7 | Erlotinib | Autophagy, EGFR | FDA approval |
| 8 | Everolimus | mTOR | FDA approval |
| 9 | Gefitinib | EGFR | FDA approval |
| 10 | Ibrutinib | BTK | FDA approval |
| 11 | Imatinib | PDGFR | FDA approval |
| 12 | Lapatinib | HER2, EGFR | FDA approval |
| 13 | Palbociclib | CDK4 | FDA approval |
| 14 | Rapamycin | Autophagy, mTOR | FDA approval |
| 15 | Ribociclib | CDK4 | FDA approval |
| 16 | Ruxolitinib | JAK | FDA approval |
| 17 | Sorafenib | Raf | FDA approval |
| 18 | Tofacitinib | JAK | FDA approval |
| 19 | Trametinib | MEK | FDA approval |
| 20 | Vemurafenib | Raf | FDA approval |
| 21 | AZD-1208 | Pim | Phase I |
| 22 | AZD-7762 | Chk | Phase I |
| 23 | JNJ-38877605 | c-Met | Phase I |
| 24 | MK-5108 | Aurora | Phase I |
| 25 | Mubritinib | HER2 | Phase I |
| 26 | PF-00562271 | FAK | Phase I |
| 27 | YM155 | BIRC5 | Phase I |
| 28 | Acadesine | AMPK | Phase II |
| 29 | Apatinib | VEGFR | Phase II |
| 30 | AZD-5363 | Akt | Phase II |
| 31 | BYL-719 | PI3K | Phase II |
| 32 | Flavopiridol | CDKs (CDK1/2/4/6/7) | Phase II |
| 33 | Refametinib | MEK | Phase II |
| 34 | Roscovitine | CDKs (CDK1/2/5) | Phase II |
| 35 | Sotrastaurin | PKC | Phase II |
| 36 | Neflamapimod | p38 MAPK | Phase II |
| 37 | AZD-4547 | FGFR | Phase II/III |
| 38 | Barasertib | Aurora K | Phase II/III |
| 39 | Alisertib | Aurora K | Phase III |
| 40 | Bardoxolone Methyl | IκB/IKK | Phase III |
| 41 | Crenolanib | PDGFR | Phase III |
| 42 | Dacomitinib | EGFR | Phase III |
| 43 | Dinaciclib | CDKs (CDK2/5/1/9) | Phase III |
| 44 | Dovitinib | FGFR, FLT3, c-Kit, VEGFR, PDGFR | Phase III |
| 45 | Ceritinib | ALK | Phase III |
| 46 | Linsitinib | IGF-1R | Phase III |
| 47 | Losmapimod | p38 MAPK | Phase III |
| 48 | Quizartinib | FLT3 | Phase III |
| 49 | Volasertib | PLK1, PLK2, PLK3 | Phase III |
| 50 | 10058-F4 | c-Myc | preclinical |
| 51 | 5-Iodotubercidin | Haspin, ADK | preclinical |
| 52 | AR-A014418 | GSK-3 | preclinical |
| 53 | EHT-1864 | Rho | preclinical |
| 54 | GNE-7915 | LRRK2 | preclinical |
| 55 | GSK429286A | ROCK | preclinical |
| 56 | KU-60019 | ATM/ATR | preclinical |
| 57 | Nocodazole | Microtubules | reference cpd |
| 58 | Vinblastine | Microtubules | reference cpd |
| 59 | Paclitaxel | Microtubules | reference cpd |
| 60 | Staurosporine | multiple kinases | reference cpd |

**Supp. Table 2. CIN-associated cDNAs source, access number, and PCR cloning oligos.**

The table shows the gene name, accession number, species of origin, and the forward (FW) and reward (RW) used oligos for PCR. The red CACC indicates the pENTR/D-topo orientation cloning sequence.

| **Gene** | **Access Number** | **Species** | **5'-sequence-3'** | |
| --- | --- | --- | --- | --- |
| BIRC5 | [BC008718](http://www.ncbi.nlm.nih.gov/entrez/query.fcgi?db=Nucleotide&CMD=Search&term=BC008718) | human | FW | CACCATGGGTGCCCCGACGT |
|  |  |  | RW | ATCCATGGCAGCCAGCTG |
| *Ccnb1* | [NM_172301.3](https://www.ncbi.nlm.nih.gov/nucleotide/NM_172301.3?report=genbank&log$=nuclalign&blast_rank=2&RID=1JC5MERV013) | mouse | FW | CACCATGGCGCTCAGGGTCAC |
|  |  |  | RW | TGCCTTTGTCACGGCCTTAG |
| *Ccnb2* | [BC008247](http://www.ncbi.nlm.nih.gov/sites/entrez?term=BC008247&cmd=Search&db=nucleotide&QueryKey=1) | mouse | FW | CACCATGGCGCTGCTCCGAC |
|  |  |  | RW | GGGGCTGCCCAGCA |
| ECT2 | [BC112086](https://www.ncbi.nlm.nih.gov/nucleotide/BC112086?report=genbank) | human | FW | CACCATGGCTGAAAATAGTGTATT |
|  |  |  | RW | TATCAAATGAGTTGTAGATCTAC |
| HEC1 | [BC035617](http://www.ncbi.nlm.nih.gov/entrez/query.fcgi?db=Nucleotide&CMD=Search&term=BC035617) | human | FW | CACCATGAAGCGCAGTTCAGTTTCCA |
|  |  |  | RW | TTCTTCAGAAGACTTAATTAGAGTAG |
| MAD2 | [BC000356](http://www.ncbi.nlm.nih.gov/entrez/query.fcgi?db=Nucleotide&CMD=Search&term=BC000356) | human | FW | CACCATGGCGCTGCAGCTCTC |
|  |  |  | RW | GTCATTGACAGGAATTTTGTAGGC |
| PRC1 | [BC003138](https://www.ncbi.nlm.nih.gov/nuccore/BC003138.1) | human | FW | CACCATGAGGAGAAGTGAGGTGCTG |
|  |  |  | RW | GGACTGGATGTTGGTTGAATTGAG |
| *Pttg1* | [BC023324](http://www.ncbi.nlm.nih.gov/sites/entrez?term=BC023324&cmd=Search&db=nucleotide&QueryKey=1) | mouse | FW | CACCATGGCTACTCTTATCTTTGTTGA |
|  |  |  | RW | AATATCTGCATCGTAACAAACAGGTG |
| *Tpx2* | [BC060619](https://www.ncbi.nlm.nih.gov/nuccore/BC060619) | mouse | FW | CACCATGTCACAAGTCCCTACTACTTA |
|  |  |  | RW | CTACTGGAACCGAGTGGAGAACT |

**Supp. Table 3. Antibodies for WB**

Table summarizing all the antibodies used for western blot detection, indicating the protein name, the vendor company and reference, the animal source and clonal type, and dilution used. (SCBT - Santa Cruz Biotechnology; CST - Cell Signaling; CNIO – Spanish National Cancer Center; BD – Becton Dickinson)

| **Protein** | **Vendor** | **Reference** | **Host** | **Dilution** |
| --- | --- | --- | --- | --- |
| AKT | CST | #9272 | Rabbit polyclonal | 1:500 |
| AKT-pSer473 | CST | #4060 | Rabbit polyclonal | 1:1000 |
| AurKA | Abcam | ab13824 | Mouse monoclonal | 1:500 |
| AurKA-pThr288 | CST | #3079 | Rabbit monoclonal | 1:500 |
| BIRC5 | Novus | NB 500-201 | Rabbit polyclonal | 1:1000 |
| Cyclin B1 | SCBT | sc-752 | Rabbit polyclonal | 1:1000 |
| Cyclin B2 | Abcam | ab185622 | Rabbit monoclonal | 1:1000 |
| ECT2 | SCBT | sc-1005 | Rabbit polyclonal | 1:500 |
| ERK | CST | #9102 | Rabbit polyclonal | 1:1000 |
| ERK-pThr202/204 | CST | #9101 | Rabbit polyclonal | 1:1000 |
| GAPDH | CNIO | FF26A | Mouse monoclonal | 1:5000 |
| HEC1 | Erich Nigg lab |  | Rabbit polyclonal | 1:2000 |
| Histone H3-pSer10 | CST | #3377S | Rabbit monoclonal | 1:1000 |
| JNK-pThr183/pTyr185 | CST | #9251 | Rabbit polyclonal | 1:500 |
| LATS1 | SCBT | sc-398560 | Mouse monoclonal | 1:500 |
| LATS1-pSer909 | CST | #9157 | Rabbit polyclonal | 1:500 |
| MAD2 | BD | 610679 | Mouse monoclonal | 1:500 |
| p38 | SCBT | sc-7972 | Mouse monoclonal | 1:500 |
| p38-pTyr182 | SCBT | sc-166182 | Mouse monoclonal | 1:500 |
| PRC1 | Abcam | ab51248 | Rabbit monoclonal | 1:1000 |
| PTTG1 | Abcam | ab3305 | Mouse monoclonal | 1:1000 |
| SRC | SCBT | sc-8056 | Mouse monoclonal | 1:500 |
| SRC-pTyr416 | CST | #6943 | Rabbit monoclonal | 1:1000 |
| STAT3-pTyr705 | SCBT | sc-8059 | Rabbit monoclonal | 1:500 |
| TAZ | CST | #4883S | Rabbit polyclonal | 1:1000 |
| TPX2 | Abcam | ab32795 | Mouse monoclonal | 1:500 |
| Vinculin | SCBT | sc-73614 | Mouse monoclonal | 1:2000 |
| YAP | Abcam | ab62751 | Rabbit polyclonal | 1:500 |
| YAP-pSer127 | CST | #4911S | Rabbit polyclonal | 1:1000 |
| YAP-pSer397 | CST | #13619 | Rabbit monoclonal | 1:500 |
| YES | GeneTex | GTX100616 | Rabbit polyclonal | 1:500 |

**Supp. Table 4. Correlation of *TPX2* expression and dasatinib sensitivity in cancer cell lines**

Table summarizing the correlation statistical data for *TPX2* mRNA expression (TPMs) and Dasatinib response (AUC), in all cancer cell lines at DepMap portal.

| **Cancer subtype** | **Number of cells** | **Pearson** | **Spearman** | **Slope** | **Intercept** | **p-value (linregress)** |
| --- | --- | --- | --- | --- | --- | --- |
| Breast | 36 | -0.548 | -0.563 | -1.84E+0 | 2.32E+1 | 5,35E-04 |
| Ovary/Fallopian Tube | 37 | -0.242 | -0.272 | -6.34E-1 | 1.35E+1 | 1,49E-01 |
| Lung | 129 | -0.115 | -0.146 | -4.84E-1 | 1.35E+1 | 1,95E-01 |
| Uterus | 27 | 0.235 | 0.255 | 1.11E+0 | 2.26E+0 | 2,39E-01 |
| Pancreas | 37 | 0.191 | 0.217 | 3.98E-1 | 7.11E+0 | 2,58E-01 |
| Bone | 13 | -0.334 | -0.352 | -1.05E+0 | 1.82E+1 | 2,65E-01 |
| Myeloid | 44 | 0.169 | 0.250 | 1.30E+0 | -3.43E-2 | 2,73E-01 |
| CNS/Brain | 44 | -0.159 | -0.102 | -3.89E-1 | 1.28E+1 | 3,04E-01 |
| Skin | 43 | -0.153 | -0.183 | -7.25E-1 | 1.66E+1 | 3,26E-01 |
| Thyroid | 9 | 0.368 | 0.383 | 5.87E-1 | 4.83E+0 | 3,30E-01 |
| Bladder/Urinary Tract | 24 | 0.200 | 0.166 | 7.75E-1 | 3.73E+0 | 3,49E-01 |
| Esophagus/Stomach | 55 | -0.118 | -0.140 | -3.71E-1 | 1.22E+1 | 3,89E-01 |
| Kidney | 21 | 0.152 | 0.191 | 5.02E-1 | 5.98E+0 | 5,11E-01 |
| Pleura | 9 | -0.201 | -0.317 | -7.38E-1 | 1.43E+1 | 6,05E-01 |
| Prostate | 4 | 0.356 | 0.800 | 7.80E-1 | 7.87E+0 | 6,44E-01 |
| Liver | 21 | 0.074 | 0.025 | 4.46E-1 | 7.01E+0 | 7,49E-01 |
| Biliary Tract | 5 | 0.174 | -0.100 | 6.87E-1 | 2.79E+0 | 7,80E-01 |
| Head and Neck | 32 | 0.045 | 0.019 | 1.16E-1 | 6.82E+0 | 8,07E-01 |
| Soft Tissue | 13 | 0.068 | 0.044 | 2.14E-1 | 8.31E+0 | 8,27E-01 |
| Lymphoid | 98 | -0.021 | 0.041 | -7.37E-2 | 9.95E+0 | 8,35E-01 |
| Peripheral Nervous System | 12 | 0.053 | 0.189 | 5.28E-1 | 7.54E+0 | 8,70E-01 |
| Bowel | 44 | -0.260 | -0.254 | -8.58E-1 | 1.61E+1 | 8,78E-01 |
